# Direct Identification of O-Glycopeptides by Low-Temperature Assisted Nanopore Technique

**DOI:** 10.34133/research.0850

**Published:** 2025-09-04

**Authors:** Jia-Hong Wang, Wenjing Ma, Zheng-Li Hu, Zhaobing Gao, Yi-Tao Long, Tiehai Li, Yi-Lun Ying

**Affiliations:** ^1^ Molecular Sensing and Imaging Center, School of Chemistry and Chemical Engineering, Nanjing University, Nanjing 210023, P. R. China.; ^2^ State Key Laboratory of Chemical Biology, Shanghai Institute of Materia Medica, Chinese Academy of Sciences, Shanghai 201203, P. R. China.; ^3^ State Key Laboratory of Drug Research, Shanghai Institute of Materia Medica, Chinese Academy of Sciences, Shanghai 201203, P. R. China.; ^4^ Chemistry and Biomedicine Innovation Center, Nanjing University, Nanjing 210023, P. R. China.

## Abstract

O-glycopeptides are highly expressed in various human cancers and play a key role in cancer progression and metastasis, making them promising biomarkers for early diagnostics. However, the inherent complexity and heterogeneity of glycans pose a major challenge for the simultaneous and precise analysis of multiple glycopeptides. Here, we developed a low-temperature nanopore technique capable of simultaneously discriminating 4 truncated O-glycopeptides with varied glycoforms. This method enables the direct identification and relative quantification of O-glycopeptides from a mixture, achieving a discrimination accuracy of 92.9%. This general strategy holds promise for the label-free analysis of glycopeptide biomarkers, with potential applications in cancer diagnostics.

## Introduction

**Fig. 1.**
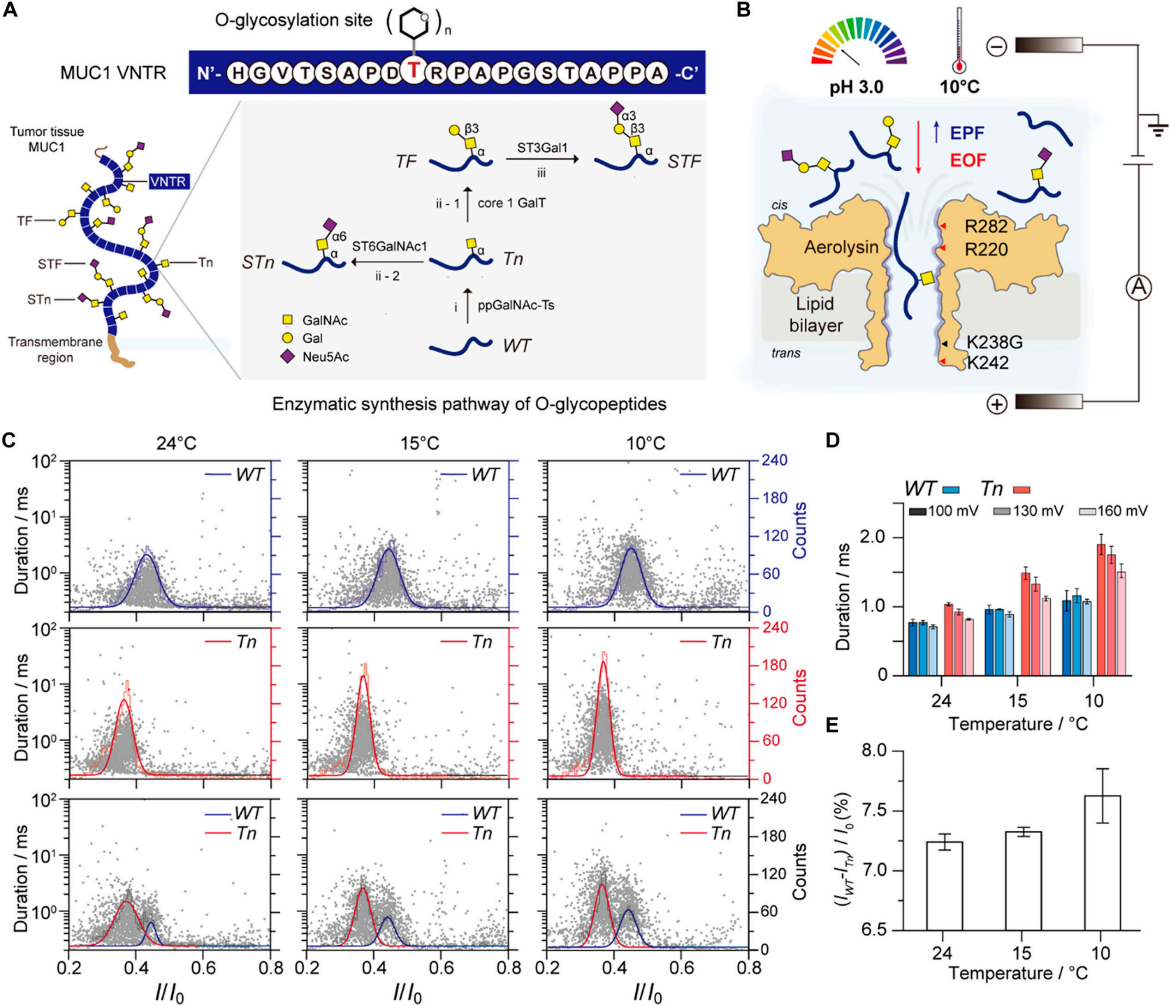
A low-temperature nanopore approach for identification of multiple O-glycopeptides. (A) Schematic diagram of truncated O-glycosylation of tumor-associated glycoprotein Mucin 1 (MUC1). MUC1 comprises the glycosylated repeat tandem region (VNTR, blue) with the sequence of N′-HGVTSAPDTRPAPGSTAPPA-C′. The enzymatic synthesis pathway of core 1 glycan (TF) at MUC1 is shown at the bottom. Firstly, a GalNAc residue is linked to a threonine (T) or serine (S) residue by polypeptide N-acetylgalactosaminyltransferases (ppGalNAc-Ts) to form the Tn antigen (GalNAcα-) via step i. Next, the Tn antigen is converted to the core 1 antigen (TF, Galβ1-3GalNAcα) by core 1 β1,3-Gal-transferase (core 1 GalT) (step ii-1) or to sialyl-Tn (Neu5Acα2-6GalNAcα-, STn) (step ii-2) by sialyltransferase ST6GalNAc1. The core 1 glycan can then be elongated to other structures or sialylated to form sialyl-TF (Neu5Acα2-3Galβ1-3GalNAcα-, STF) (step iii) by α2,3-sialyltransferases (ST3Gal1). (B) Schematic representation for the detection of single glycopeptide using the K238G Aerolysin at pH 3 and 10 °C. The bias potential is applied using Ag/AgCl electrodes, and the *cis* compartment is defined as a virtual ground. The negatively charged amino acids of the nanopore are prone to be protonated at pH 3, exposing the positively charged amino acids of R282, R220, and K242 at the lumen (red triangle). The lysin (K) is mutated to glycine (G) at the 238 site (black triangle). Under a positive applied potential, the electrophoretic force (EPF) and electroosmotic flow (EOF) acting on the peptide are in opposite directions. The experimental details are shown in Materials and Methods. (C) Scatterplots of *I*/*I*_0_ versus duration and histograms of *I*/*I*_0_ for WT (upper row), Tn (middle row), and mixture of WT and Tn (bottom row) at temperatures of 24 °C (left), 15 °C (middle), and 10 °C (right). *I* and *I*_0_ represent the blockage current and open-pore current, respectively. The blue and red curves represent the Gaussian fitting to the *I*/*I*_0_ histograms for WT and Tn peptides, respectively. The light blue line represents the outline of the histogram of WT, and the light red line represents the outline of the histogram of Tn. The gray line represents the outline of the histogram of mixture. (D) Temperature dependence of the duration for WT (blue) and Tn (red) peptides at various voltages. (E) Temperature dependence on the current peak separation of (*I*_WT_ − *I*_Tn_)/*I*_0_. The concentration of WT and Tn peptides is fixed at 10 μM, and the electrolyte solution was 1 M KCl, 10 mM tris, and pH 3. Error bars depict the standard deviation (SD) of 3 independent measurements.

Protein glycosylation is a complicated posttranslational modification that mediates a diversity of cellular processes [1–3]. Mucin-type (GalNAc type) O-glycosylation is one of the most diverse glycosylation of secretory and membrane-bound proteins. Its alteration serves as a hallmark of cancer that has helped to shape the management and understanding of cancer [4]. Typically, the tumor-associated glycoprotein Mucin 1 (MUC1) is commonly overexpressed in various epithelial adenocarcinomas such as lung, liver, colon, breast, pancreatic, and ovarian cancers [5,6]. It comprises a highly variable number of glycosylated repeat tandem regions (VNTRs), a transmembrane region, and other segments [7]. Due to reduced glycosyltransferase activity with simultaneously increased sialyltransferase activity in the tumor tissue, the O-glycosylation of MUC1 tends to be altered from long branched chains to short structures and capped with terminal sialic acids [8]. This process is known as truncated O-glycosylation (Fig. 1A). A representative truncated mucin-type O-glycan is the Thomsen–Friedenreich antigen (also known as core 1, TF), which forms the core of many long and complex structures [9]. The biosynthetic pathway of TF is as follows: First, a GalNAc residue is linked to a serine or threonine residue by polypeptide N-acetylgalactosaminyltransferases (ppGalNAc-Ts) to form Tn antigen (GalNAcα-) (Fig. 1A, step i). Next, the Tn antigen is further converted to TF (Galβ1-3GalNAcα) or sialyl-Tn (Neu5Acα2-6GalNAcα-, STn), which is catalyzed by the enzyme core 1 β1,3-Gal-transferase (core 1 GalT) or sialyltransferase ST6GalNAc1, respectively (Fig. 1A, step ii). After that, TF can be further elongated into a series of glycan structures, or be sialylated to form sialyl-TF (Neu5Acα2-3Galβ1-3GalNAcα-, STF), which is catalyzed by the enzyme α2,3-sialyltransferases (ST3Gal1) (Fig. 1A, step iii) [8]. Recognition of multiple glycosylated variants of peptides is highly required for reliable cancer diagnostics and prognosis. Strategies for glycopeptide detection include enzymatic or chemical cleavage methods to release glycans from glycopeptides [10,11]. The enzymatic methods are generally selective for certain specific glycan linkages or glycoprotein substrates, whereas chemical methods (e.g., base-mediated β-elimination) may cause unintended side reactions, complicating glycan analysis [12]. Moreover, these approaches often require costly enzymes or careful handling of reactive chemicals. As an alternative, direct characterization of glycopeptides offers valuable insights into site-specific glycosylation, commonly carried out by liquid chromatography–tandem mass spectrometry (LC-MS/MS) or immunoassays [2,13]. For mass spectrometry, ionization and detection efficiency of glycopeptides vary with their chemical properties, leading to potential uncertainty. For instance, sialic acid modifications at glycan terminal can result in lower mass intensity compared to other glycopeptides at equimolar concentrations [14]. Immunoassays, on the other hand, require antibody or lectin labeling, and may be influenced by factors such as glycan binding affinity and potential cross-reactivity with structurally similar glycans [15]. Given these considerations, there remains a need for approaches that enable the direct identification of O-glycopeptides in a label-free manner.

Nanopore-based single-molecule sensing utilizes the measurement of ionic current flow caused by individual analytes to detect their structure, composition, and length. Due to the confined space, nanopores exhibit high sensitivity in analyzing DNA [16], peptides [17–23], proteins [24–27], oligosaccharides [28–33], and other small molecules [34,35]. This approach offers the advantages of being label-free and enabling rapid detection. However, there are still challenges in detecting glycopeptides with nanopore techniques. Unlike posttranslational modifications such as phosphorylation, acetylation, and methylation, glycosylation involves larger and more complex chemical structures [36]. Recently, only few studies have reported nanopore-based glycoprotein detection [37–39], with most focusing on identifying a monosaccharide modification on peptides (e.g., glucose). Glycopeptides bearing oligosaccharide modifications (such as disaccharides or trisaccharides) face larger entropic barriers during nanopore translocation compared to other posttranslational modifications (e.g., phosphorylation or acetylation), due to the greater steric hindrance and conformational restrictions imposed by the bulky glycan moieties. Additionally, glycopeptides differing by a single glycan residue often generate similar ionic current events, making them more challenging to distinguish using nanopore sensing. To address these challenges, we developed a general strategy using a low-temperature and low-pH aerolysin nanopore technique to enhance the capture capability for O-glycopeptides with monosaccharide to trisaccharide modifications. It also extends the interaction time inside the nanopore and thereby improves the discrimination ability of varied O-glycopeptides. This method enables direct, label-free identification and quantification of O-glycopeptides, achieving a 92.9% discrimination accuracy, and holds promise for potential application in cancer diagnostics.

## Results and Discussion

### Fine-tuning temperature and pH conditions for single O-glycopeptide detection

As a proof of concept, herein, we selected truncated O-glycosylated variants (Tn, STn, TF, STF) of MUC1 peptides (Fig. 1A). Considering the neutral net charge of the MUC1 peptides, we first employed N226Q/S228K aerolysin (AeL) due to its good performance in the capture and sensing of neutrally charged peptides [40]. All experimental details and characterization of glycopeptides and nanopores are shown in Materials and Methods and Figs. S1 to S3. The wild-type (WT) peptide can be captured by N226Q/S228K AeL. However, the WT peptide exhibited relatively short event durations, with a slight increase observed as the voltage increased (Figs. S4 and S5). Structural prediction indicated that residues 11 to 15 of the WT MUC1 peptide may form a loop structure (Fig. S6). This is likely due to proline, which introduces a kink in the backbone and promotes β-turn formation [41–43]. Since N226Q/S228K AeL shows a small diameter of around 0.8 nm at its constriction [40], the partially folded structure of the peptides may hinder their translocation through the nanopore. Compared to N226Q/S228K AeL, K238G AeL shows a relatively larger diameter of around 1.1 nm at the constricted region [44], making it potentially more suitable for O-glycopeptide detection. However, K238G AeL hardly captures the WT peptide in pH 7.4, due to its weak electroosmotic flow in the inward direction (from *cis* to *trans*) (Fig. S7). To detect the neutrally charged and partially folded MUC1 peptides, we employed the K238G AeL nanopore under acidic conditions (pH 3) to enhance electroosmotic flow (EOF). At low pH, glutamate and aspartate residues are protonated, leading to the disruption of interactions between paired acidic and basic residues (R282:D216, R220:D222, K238:E258, K242:E254) [21,45,46], which in turn strengthens EOF and facilitates peptide capture (Fig. 1B and Fig. S8). Acidic conditions may also disrupt intramolecular interactions within the folded peptide [47], making the peptide more prone to be captured by the nanopore. As shown in Fig. 1C, MUC1 peptides are effectively captured at pH 3 with the K238G AeL, due to the protonation-induced exposure of up to 21 positively charged residues in the heptameric K238G AeL under acidic conditions, thereby further enhancing EOF for peptide capture efficiency (Table S1).

To demonstrate the versatility of this approach, we further detected the Tn peptide, which was N-acetylgalactosaminylated. Due to the larger size of Tn, it causes a deeper current blockage than the unmodified WT peptide (Fig. 1C). The study on voltage dependence showed that the WT peptide could traverse the K238G nanopore at voltages above +160 mV, at pH 3 and 24 °C (Fig. 1D). Interestingly, the voltage dependence revealed that N-acetylgalactosaminylation of MUC1 peptides facilitates their passage through the nanopore. To further validate the discrimination ability of this method, a mixture of WT and Tn was analyzed. As shown in Fig. 1C, the events of WT and Tn fall into two Gaussian distributions of *I*/*I*_0_, with WT located at *I*/*I*_0_ = 0.44 and Tn at *I*/*I*_0_ = 0.37. However, the peak separation needs to be further improved due to wide and overlapping event clusters. Previous studies showed that the prolonged duration of the nanopore blockade would lead to a narrower *I*/*I*_0_ distribution with high peak separation [48,49]. Therefore, we further lowered the solution temperature from 24 °C to 10 °C to prolong the duration. The results showed that the durations of WT and Tn peptides increased by 1.5 and 1.8 times at +160 mV, respectively (Fig. 1D). As a result, at 10 °C, the peak separation between WT and Tn was improved compared to that at 24 °C (Fig. 1E and Figs. S9 and S10), proving that lowering temperature enhances the sensing ability of nanopores. Interestingly, at 10 °C, the duration of the Tn peptide showed a decrease with increasing voltage, consistent with the trend observed at 24 °C (Fig. 1D). These results suggest that Tn peptides may translocate across the nanopore even at lower temperatures. In contrast, the WT peptide demonstrated limited sensitivity to voltage changes across the examined temperature range. The temperature-dependent experiments further revealed the enthalpic and entropic changes associated with peptide–nanopore interactions (Fig. S11). Compared to the unmodified peptide, the Tn peptide modified with N-acetylgalactosamine exhibited a greater enthalpy change and a lower entropy change inside the nanopore. Moreover, the capture frequency of Tn is positively correlated with the applied voltage. The high voltage of +160 mV also results in a good signal-to-noise ratio of 17.3 (Fig. S12 and Table S2). Thus, the optimal measurement conditions for distinguishing the O-glycopeptides with K238G AeL are +160 mV, 10 °C, and pH 3. The low-pH and low-temperature activated K238G AeL not only enhances the capture capability but also strengthens the interaction with the glycopeptides. Note that the open-pore current is stable for about at least 100 min at this experimental condition, ensuring the long-time and stable sensing (Fig. S13).

### Identification of various truncated O-glycopeptides

**Fig. 2.**
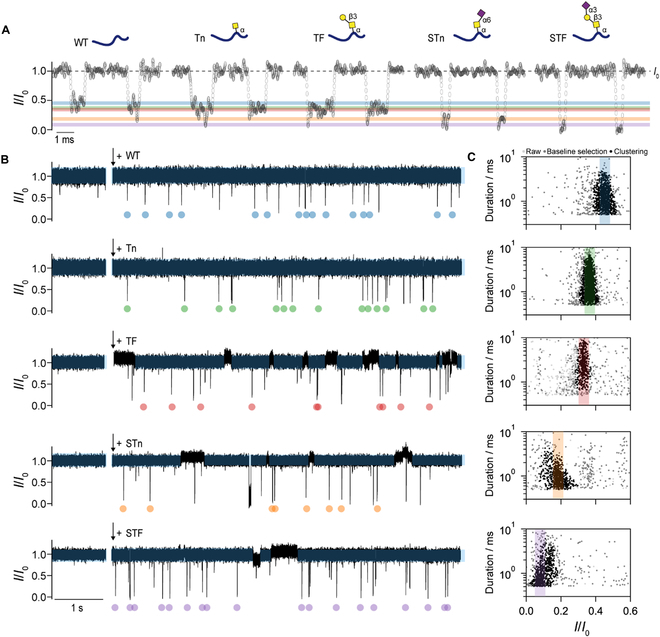
Identification of truncated O-glycopeptides with K238G AeL at 10 °C. (A) Representative raw events of WT, Tn, TF, STn, and STF peptides, respectively (from left to right). (B) Typical current traces before and after addition of WT, Tn, TF, STn, and STF peptides (from top to bottom). The selected range of baseline is shown in blue band covering the current trace. The colored dot labels the characteristic event from the related truncated O-glycopeptides. (C) Scatterplots of *I*/*I*_0_ versus duration. The light gray, gray, and black dots represent the raw events, events after baseline selection, and events after clustering of the truncated O-glycopeptides corresponding to (B). The center of color bands highlights the statistical peak *I*/*I₀* values obtained from Gaussian fitting (see Fig. S16), which are 0.45 for WT (blue), 0.36 for Tn (green), 0.33 for TF (pink), 0.18 for STn (orange), and 0.10 for STF (purple), respectively. Gray scatter points may result from baseline fluctuations due to O-glycopeptide–bilayer interactions and/or trace impurities arising from the synthetic complexity of larger glycans. The experiments were performed in 1 M KCl, 10 mM tris, and pH 3 under an applied voltage of +160 mV.

Subsequently, we employed this method to detect 4 types of truncated O-glycopeptides with different modifications, which are Tn, TF, STF, and STn. As shown in Fig. 2A and B, all the O-glycopeptides could be efficiently captured by K238G AeL and produced distinct blockage events. Since O-glycopeptides could interact with the bilayer [50] (Fig. S14), the open-pore current shows slight fluctuations following nanopore insertion and peptide addition, especially for TF and STn. Therefore, Gaussian fitting was applied to evaluate baseline noise and minimize its interference in the statistical analysis of single-peptide events (Fig. S15). Subsequently, we clustered the events using the HDBSCAN algorithm to remove outliers and noise (Fig. S16, Materials and Methods). The results showed that STn and STF peptides showed 2 population in scatterplots, which could be attributed to the electrostatic interactions between the sialic acid carboxyl group of the peptide and arginine residues inside the nanopore, similar to the results from sialylated glycans in α-hemolysin [31]. This difference may also result from conformational changes of the glycopeptides inside the nanopore. As shown in Fig. 2C, these 5 peptide variants differ in both blockage current and duration, demonstrating the distinguishability of the activated K238G AeL. The shorter durations of sialylated glycans (STn and STF) may be attributed to their partially negative charge at pH 3, which likely enhances electrophoretic transport under +160 mV.

### Discrimination of O-glycopeptides by machine learning

**Fig. 3.**
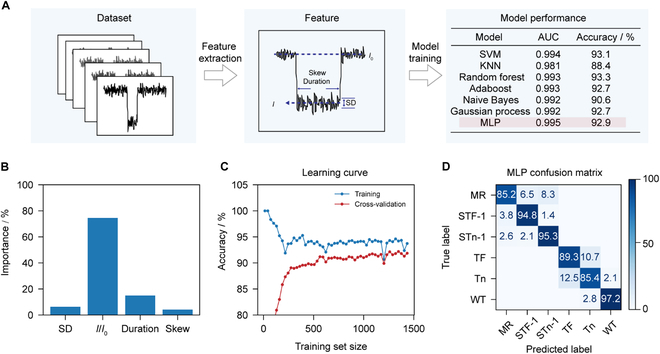
Identification of various O-glycopeptides by machine learning. (A) Workflow of the machine learning. A total of 2,400 events in the dataset were used, which include WT, Tn, TF, STn-1, and STF-1 events and mix events. Because both STn and STF events showed 2 distributions in the scatterplot, the STn and STF events were clustered into 2 classes, labeled STn-1, STn-2 and STF-1, STF-2, respectively. The STn-2 and STF-2 events, which show substantial overlap, were merged into a single class called merging region (MR) events. Events from the MR class were ignored during statistics. More details of the data analysis and feature definition can be found in Materials and Methods and Fig. S17. Four features (SD, skewness, duration, and *I*/*I*_0_) were extracted from ionic current events to form a feature matrix. Here, SD represents the standard deviation of the current amplitude within the indicated range of the event, while Skew refers to its skewness. Seven models were employed separately, and their corresponding AUC values and test set accuracies were reported. Among these, the multilayer perceptron (MLP) model performed the best. (B) Importance of 4 features (SD, *I*/*I*_0_, duration, and skewness) in model training. (C) Learning curve of the MLP model produced by performing model training and validation using varying sample size. (D) Confusion matrix for the classification of various truncated O-glycopeptides generated by the MLP model.

To further achieve automatic identification of 4 types of truncated O-glycopeptides, we employed a supervised machine learning algorithm to recognize these peptides (Materials and Methods and Fig. 3). The entire machine learning workflow includes data acquisition, feature extraction, and model training (Fig. 3A). A total of 2,400 events from 5 pure peptide samples detected by K238G AeL were collected. From these blockade events, 4 statistical features were extracted to form the feature matrix, which are *I*/*I*_0_, duration, standard deviation (SD), and skewness. Subsequently, 80% of the dataset was extracted as the training set and 20% as the test set.

To prevent overfitting, 10-fold cross-validation was used during model training and validation. We trained 7 models, including support vector machine (SVM), k-nearest neighbors (KNN), Random forest, Adaboost, Naïve Bayes, Gaussian process, and multilayer perceptron (MLP), and then evaluated their classification performance based on the area under the receiver operating characteristic curve (AUC) and accuracy (Fig. 3A). Among these models, the MLP model performed the best, achieving the highest AUC (0.995) and relatively high accuracy (92.9%) on the test set. The feature contribution proved that *I*/*I*_0_ contributed the most to discrimination (74.5%), while the contribution of SD, duration, and skewness are relatively slight, which are 6.3%, 15.0%, and 4.2%, respectively (Fig. 3B). Additionally, the learning curve demonstrated that the model’s accuracy reached 92.6% at 570 events, proving that our data size is entirely sufficient (Fig. 3C). The calibration curves align closely with the diagonal, and both the precision–recall (average precision > 0.93) and receiver operating characteristic (AUC > 0.98) curves show good performance for all peptide classes (Fig. S18). The confusion matrix generated by the MLP model showed excellent performance in classifying all classes, and the highest true positive rate is 97.2% for WT peptide.

### Relative quantification in the mixture of various O-glycopeptides

**Fig. 4.**
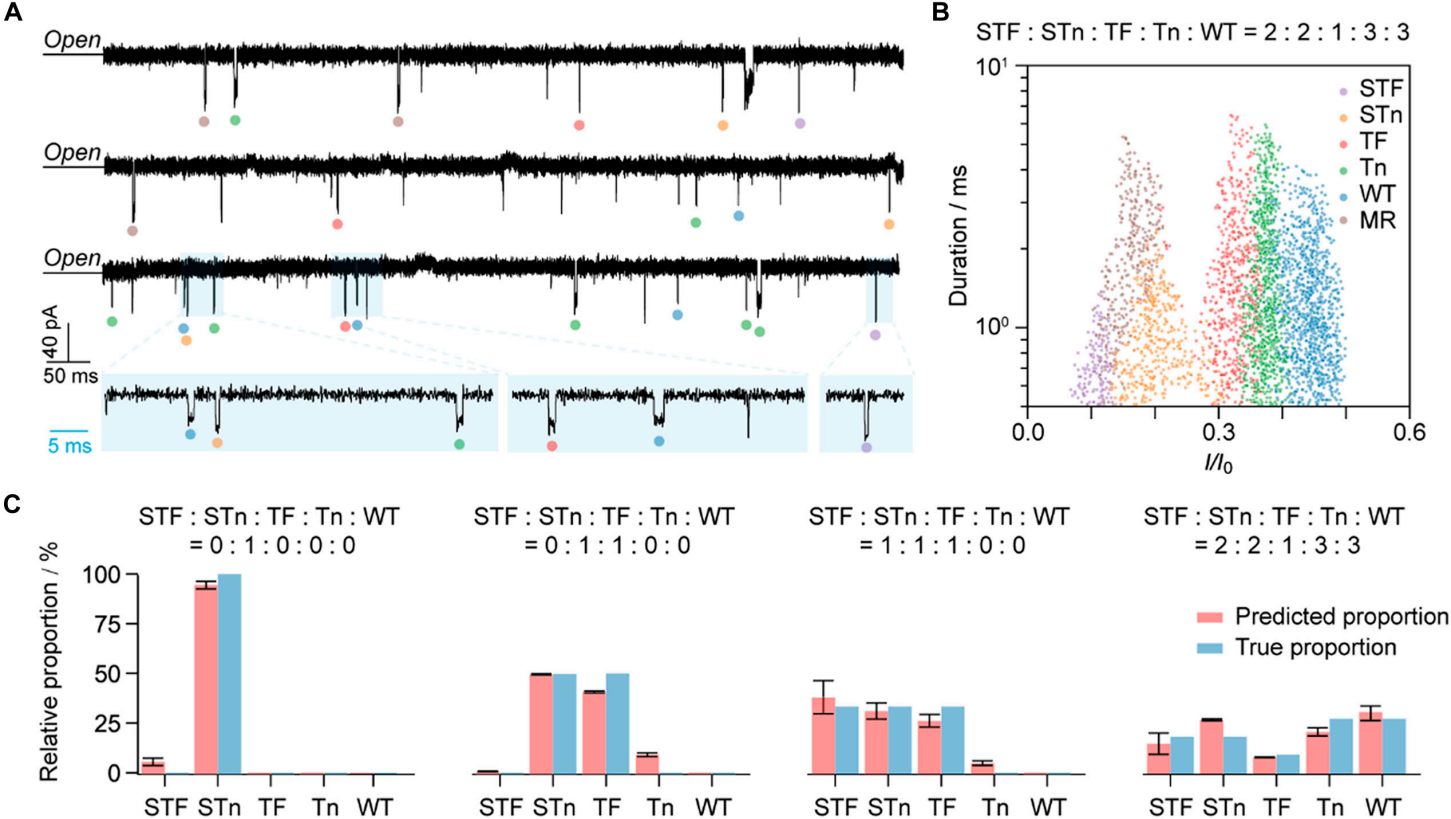
Validation of the effectiveness of developed machine learning model. (A) Current traces of the mixture composed of 5 types of O-glycopeptides. The labels were automatically generated by the MLP model. The blue area represents the zoom-in view of the current trace. All current traces are obtained from the same experiment. (B) Scatterplots of *I*/*I*_0_ versus duration of the mixture corresponding to (A). The label was predicted by the MLP model. The blockade ratio (*I*/*I₀*) and its statistical SD for each predicted peptide in the mixture were listed in Table S4. (C) Comparison of the predicted proportion and true proportion of the various peptides in mixtures. The molar proportions of these analytes were STF:STn:TF:Tn:WT of 0:1:0:0:0, 0:1:1:0:0, 1:1:1:0:0, and 2:2:1:3:3 (from left to right), respectively. The measurement was performed in 1 M KCl, 10 mM tris, and pH 3 under an applied voltage of +160 mV at 10 ± 1 °C.

To validate the effectiveness of the machine learning model, we tested mixtures with different molar ratios. The current traces and scatterplots of the mixtures composed of 5 types of peptides are shown in Fig. 4A and B. The trained model, incorporating the capture efficiency of pure peptides, allows identification and relative quantification of the glycopeptides (Fig. 4C, Materials and Methods, and Table S3). As shown in Fig. 4C, the proportions predicted by the machine learning model were consistent to the true proportions for all mixtures. In particular, STn, a clinical cancer biomarker [51], showed recoveries of 99.4% and 93.3% in the STF:STn:TF:Tn:WT mixtures (0:1:1:0:0) and (1:1:1:0:0), respectively (Fig. 4C). The minor difference in the predicted proportion and true proportion may be attributed to the potential competitive capture among these substances present. To validate the stability of this method in a complex background, we analyzed O-glycopeptides in 0.2% cell lysate and 1% bovine serum albumin (BSA), respectively. The scatterplots from the machine learning predictions demonstrated that the method could identify the components of the mixture (Figs. S19 to S21). These glycosylated variants have been reported with varying expression levels across different types of cancers [52,53]. Our method can recognize and quantify these variants in complex mixtures and may help identify these glycopeptides in physiological samples.

## Conclusion

In summary, we developed a highly sensitive and label-free nanopore strategy for the identification and relative quantification of multiple O-glycopeptides. This versatile approach utilizes low-pH and low-temperature conditions to activate K238G AeL, enhancing its capture and sensing capabilities for the direct detection of glycopeptides. Notably, glycopeptides can be identified within just 20 min of recording. The accuracy of the machine learning model reaches 92.9%, with an AUC value of 0.995. The development of nanopore array chip [54] could enable high-throughput single-molecule sensing of complex glycopeptide variants, opening up potential applications for glycosylation-based biomarker detection.

To enable practical applications, it is critical to address the complexity of sample pretreatment and ensure reproducibility in real biological samples. Moreover, the reported concentrations of truncated O-glycosylated peptide variants in serum are around the nanomolar range, posing a challenge for nanopore detection sensitivity [55]. A possible solution involves integrating MUC1-specific antibody-based enrichment prior to nanopore detection, allowing for the selective isolation of target glycopeptides from complex biological matrices. Additional optimization is likely required to integrate the system into a filter membrane-based device for reliable sample analysis. To better analyze longer and more complex glycan chains, we are exploring enzymatic or chemical cleavage methods to release and individually detect glycans and peptides using aerolysin nanopore. Future advancements, such as de novo protein design [56], may help develop elegant nanopore interfaces capable of recognizing larger glycan branches and more intricate glycopeptides. Since the glycosylation of MUC1 involves multiple glycosyltransferases, real-time monitoring of glycopeptide substrates and products by nanopore sensing may provide useful insights into enzymatic pathways. When combined with nanopipette-based single-cell analysis, this approach could potentially be applied to probing glycosyltransferase activity and recognition of the glycopeptide at the single-cell level.

## Materials and Methods

### Reagents and materials

Tris (hydroxymethyl) aminomethane (tris, ≥99.9%), hexadecane, hexane, trypsin-agarose, BSA, potassium chloride (≥99%), and decane (anhydrous, ≥99.8%) were purchased from Sigma-Aldrich Co. Ltd. (St. Louis, USA). 1,2-Diphytanoyl-sn-glycero-3-phosphocholine (powder) was purchased from Avanti Polar Lipids Inc. (Alabaster, USA). Hydrochloric acid (HCl) was purchased from Nanjing Chemical Reagent Co. Ltd. (Jiangsu, China). Radioimmunoprecipitation assay (RIPA) lysis buffer (moderate) was purchased from Beyotime (Shanghai, China). Sodium dodecyl sulfate–polyacrylamide gel electrophoresis analysis (SDS-PAGE) preparation kit was purchased from Sangon Biotech Co. Ltd. (Shanghai, China). To facilitate the formation of the lipid bilayer, the preparation solution was made by mixing hexadecane and hexane with a volume ratio of 1:99. Phospholipid solution with a concentration of 30.0 mg/ml was obtained by dissolving phospholipids in decane. The truncated mucin-type O-glycopeptides were synthesized according to our previous work [57]. The LC-MS characterizations are shown in Fig. S3. The concentrations of glycopeptides in the pure sample experiment were fixed at 10 μM (WT), 10 μM (Tn), 2 μM (TF), 5 μM (STn), and 3 μM (STF), respectively. In the mixture of STF:STn:TF:Tn:WT, the glycopeptide concentrations are as follows: 2 μM STn and 2 μM TF for the 0:1:1:0:0 mixture; 2 μM (STF), 2 μM (STn), and 2 μM (TF) for the 1:1:1:0:0 mixture; 2 μM (STn), 2 μM (STF), 2 μM (STn), 1 μM (TF), 3 μM (Tn), and 3 μM (WT) for the 2:2:1:3:3 mixture. The pH of the acidic buffer solution was measured using a pH meter (LE422, Mettler-Toledo, UK; accuracy ± 0.01) after the addition of 10 μl glycopeptides solution, yielding values of 3.00 at 24 °C, 3.04 at 15 °C, and 3.07 at 10 °C under homebuilt temperature controller (accuracy ± 1 °C). Unless noted, all solutions in this work were prepared with ultrapure water (18.2 MΩ·cm at 25 °C) obtained using a Milli-Q system (Massachusetts, USA). For cell extraction, about 10^6^ gliacyte cells were collected and resuspended in 500 μl of 1× phosphate-buffered saline buffer. Then, the cells were pelleted and resuspended in ice-cold RIPA lysis buffer. After that, the cell lysate was centrifuged at 10,000 rpm for 5 min to remove debris and insoluble material. The supernatant was then continuously centrifuged by Amicon 30 K Ultra Centrifugal Filter Device (Merck KGaA, Darmstadt, Germany). Therefore, the molecules with a molecular weight of less than 30 kDa were almost excluded from the lysate. Then, the lysate was ready for nanopore experiments.

### Proaerolysin preparation and characterization

Proaerolysin was prepared according to our previous work [58]. We reconstructed the K238G and N226Q/S228K mutant expression plasmids by polymerase chain reaction and transformed them into *Escherichia coli* BL21 (DE3) pLysS cells. Protein expression was induced by adding isopropyl-β-d-thiogalactoside to a 0.5 mM final concentration at 16 °C for 6 h. The collected K238G and N226Q/S228K proaerolysin proteins were characterized by 12% SDS-PAGE (Fig. S1). To obtain the activated monomers, proaerolysin was pretreated with a trypsin solution at a mass ratio of 10:1 (trypsin to proaerolysin) for 6 h at room temperature.

### Single-channel recording

Nanopore experiments were performed according to our previous work [58]. The homemade nanopore sensing device consists of 2 compartments that are separated by a Teflon sheet (FP301200, Goodfellow Cambridge Ltd., UK), which contains an aperture of ~50 μm in diameter for lipid membrane formation. The 2 compartments are termed as the *cis* chamber and the *trans* chamber, where the *cis* chamber is grounded. Firstly, the aperture was pretreated with hexadecane solution for 20 min, and then ~1.5 μl of phospholipid solution was added to the surface of electrolyte solutions in both chambers. After the phospholipid is evenly dispersed on the solution surface, the air–solution interface is moved below and above the aperture using a syringe device to form a lipid bilayer. When a mechanically stable lipid membrane is obtained, approximately 1.0 μl of activated proaerolysin solution is injected near the aperture into the *cis* chamber. Once the insertion of a single AeL was observed, the analytes were added into the *cis* chamber. Then, the voltage was adjusted to +100, +130, and +160 mV, respectively, and the corresponding ionic current traces were recorded. The experimental temperature was controlled by a home-made temperature device. The nanopore currents were collected via a pair of Ag/AgCl electrodes by the nanopore instrument Cube-D2 (https://zenodo.org/records/11609574), and all data were recorded with a low-pass filter of 5 kHz and a sampling frequency of 100 kHz. The K238G AeL was characterized by *I*–*V* curves (Fig. S2).

### Feature extraction and statistical analysis

The single-molecule ionic current events were extracted and analyzed by the software “PyNanoLab” (https://doi.org/10.5281/zenodo.11383973). In the statistical analysis, the *I*/*I*_0_ values were extracted from the Gaussian fits of the residual current blockage (*I*/*I*_0_) histograms, where *I* is the blockage current and *I*_0_ is the open-pore current. The events with duration time less than 0.50 ms were excluded for minimizing the evaluation errors.

The average open-pore current (*I*_0_) and SD of baseline (σ) for each recording were determined statistically, and a threshold of *I*_0_ − 5σ was used to define events for the target peptides in the experiments. The statistical duration was obtained by the Gaussian fitting of the log(duration) histogram. The peak separation is defined as (*I*_WT_ − *I*_Tn_)/*I*_0_. The skewness is a measure of the asymmetry of a data distribution; its calculation formula is as follows:

$$\text{Skewness}=\frac{\frac{1}{n}\sum_{i=1}^{n}{\left(x_{i}-\bar{x}\right)}^{3}}{{\left(\sqrt{\frac{1}{n}\sum_{i=1}^{n}{\left(x_{i}-\bar{x}\right)}^{2}}\right)}^{3}}$$ (1)

where *n* represent the number of samples and *x_i_* represents the value of every sample. From each event, 5 event features, that is, *I*/*I*_0_, SD, skewness and duration, were extracted using the software “PynanoLab”. The feature importance was calculated using the permutation importance method, which evaluates the impact of each feature on the model’s predictive performance by measuring the change in accuracy when the feature values are randomly permuted [59]

According to the Eyring equation (Eq. 2), a linear fit of ln(*k*_off_/*T*) versus 1/*T* allows for the calculation of the enthalpy (*ΔH*) and entropy (*ΔS*) changes for the interactions between the nanopore and analytes, based on the slope and intercept of the fitted line:

$$\begin{array}{c} \ln\left(\frac{k_{\mathrm{off}}}{T}\right)=-\frac{\Delta H}{R}\cdot \frac{1}{T}+\ln\left(\frac{k_{B}}{h}\right)+\frac{\Delta S}{R} \end{array}$$ (2)

where *k*_off_ = 1/τ_off_, *T* is the experimental temperature, *k*_B_ is the Boltzmann constant, *h* is the Planck constant, and *R* is the gas constant. As shown in Fig. S11, the Tn and WT peptides exhibit distinct temperature-dependent behaviors. The Tn peptide shows higher enthalpy (47.74 kJ/mol) and lower entropy (–20.3 J/mol·K) changes than the WT peptide (25.89 kJ/mol and –95.2 J/mol·K), indicating that the N-acetylgalactosamine modification enhances the interaction strength with the nanopore.

### Clustering analysis by HDBSCAN algorithm

To remove noises from nanopore gating and interaction between peptide and the membrane, an HDBSCAN algorithm was used for cluster analysis of the recorded events. To preserve the most clustered population in the scatterplot of the *I*/*I*_0_ versus duration, the min cluster size and the min samples were adjusted to appropriate values. The data before and after cluster analysis are shown in Fig. S16. In the comparison, the distribution of the events is similar, confirming the reliability of the analysis.

Both STn and STF show 2 distributions *I*/*I*_0_ (Fig. S17). Thus, the events of STn and STF are clustered into 2 types by Agglomerative Clustering algorithm, which are STn-1, STn-2 and STF-1, STF-2, respectively. Then, the events of STn-2 and STF-2 are combined into one class named “merging region” (MR). Events from this class would be ignored for further statistical analysis, because they are more dispersed and highly overlapping (Fig. S17). The HDBSCAN algorithm and Agglomerative Clustering algorithm come from scikit-learn, performed with Python 3.10.

### Machine learning

The dataset contains a total of 2,400 events, composed of 6 classes: WT, Tn, TF, STn-1, STF-1, and MR. The input data were randomly split into a training set (80% of the labeled dataset) and a testing set (20%) for model training and model testing. The data in the training set were first standardized and then applied to train 7 models, including SVM, KNN, Random forest, Adaboost, Naïve bayes, Gaussian process, and MLP. According to the 10-fold cross-validation accuracy, the models with the highest AUC value and accuracy were selected. A learning curve of the best model for varying training sample size was generated to evaluate whether the amount of data is sufficient and to determine if the model is overfitting. The feature importance was calculated using the permutation importance method, which evaluates the impact of each feature on the model’s predictive performance by measuring the change in accuracy when the feature values are randomly permuted. A confusion matrix was generated using the testing set for model evaluation. All models come from scikit-learn, performed with Python 3.10.

The relative quantification values obtained by machine learning will be corrected based on the capture frequency of pure peptide samples. The results are shown in Table S3.

## Data Availability

All data supporting the findings of this study are included within the article and its Supplementary Materials, as well as from the corresponding author upon reasonable request.
