# Supplementary material for: Direct Identification of O-Glycopeptides by Low-Temperature Assisted Nanopore Technique
Source: Research (Wash D C). 2025 Sep 4;8:0850. doi: 10.34133/research.0850 (PMC12410930; doi:10.34133/research.0850)
Supplement: Supplementary 1 — Figs. S1 to S21 Tables S1 to S4 [file research.0850.f1.docx]

**Supporting Information**

Direct Identification of O-Glycopeptides by Low-Temperature Assisted Nanopore Technique

Jia-Hong Wang^1^†, Wenjing Ma^2^†, Zheng-Li Hu^1^, Zhaobing Gao^3*^, Yi-Tao Long^1^, Tiehai Li^2*^and Yi-Lun Ying^1,4*^

^1^Molecular Sensing and Imaging Center, School of Chemistry and Chemical Engineering, Nanjing University, Nanjing 210023, P. R. China.

^2^State Key Laboratory of Chemical Biology, Shanghai Institute of Materia Medica, Chinese Academy of Sciences, Shanghai 201203, P. R. China. ^3^State Key Laboratory of Drug Research, Shanghai Institute of Materia Medica, Chinese Academy of Sciences, Shanghai 201203, P. R. China. ^4^Chemistry and Biomedicine Innovation Center, Nanjing University, Nanjing 210023, P. R. China.

^*^Address correspondence to: yilunying@nju.edu.cn (Y.-L. Y.); tiehaili@simm.ac.cn (T. L.); zbgao@simm.ac.cn (Z. G.);

†These authors contributed equally to this work.

**Abstract**

O-glycopeptides are highly expressed in various human cancers and play a key role in cancer progression and metastasis, making them promising biomarkers for early diagnostics. However, the inherent complexity and heterogeneity of glycans pose a major challenge for the simultaneous and precise analysis of multiple glycopeptides. Here, we developed a low-temperature nanopore technique capable of simultaneously discriminating 4 truncated O-glycopeptides with varied glycoforms. This method enables the direct identification and relative quantification of O-glycopeptides from a mixture, achieving a discrimination accuracy of 92.9%. This general strategy holds promise for the rapid, label-free analysis of glycopeptide biomarkers, with potential applications in cancer diagnostics.

**Table of Contents**

**Fig. S1.** SDS-PAGE analysis of nanopores……………………………………………………………………………………………………………….S3

**Fig. S2.** *I*-*V* curves of the K238G AeL at 24 ± 1°C and 10 ± 1°C………………………………………………...................................S4

**Fig. S3.** The LC-MS characterization of truncated O-glycopeptides………………………………………..…..............................S5

**Fig. S4.** Typical current trace and the scatter plot of *I*/*I*_0_ vs duration of WT peptide detected by N226Q/S228K AeL………………………………………………………………………………………………….........................................................................S6

**Fig. S5.** The voltage dependency of duration of the WT peptide detected by N226Q/S228K AeL…..………………………S7

**Fig. S6.** The structure of WT peptide……………………………………………………………………..…...……………………………………..….S8

**Fig. S7.** Typical current trace of WT peptide detected by K238G AeL at 24 ± 1 °C and pH 7.4………….…………………..…S9

**Fig. S8.** Typical current trace and the scatter plot of *I*/*I*_0_ vs duration of WT peptide detected by K238G AeL at 24 ± 1 °C and pH 3………………………………………………………………………………………………………………………………………………….……S10

**Fig. S9.** Scatter plots of pure WT, Tn and their mixture.……………………….……………………………......................................S11

**Fig. S10.** Histograms of pure WT, Tn and their mixture…………………….…………………..…………………………………………………S12

**Fig. S11.** The scatter plot of ln (k_off_/T) versus 1/T of the WT and Tn peptides……….……………………….………………………S13

**Fig. S12.** Signal-noise ratio (SNR) boxplot of WT peptide detection using K238G AeL under different temperatures and voltages.…………………………………………………………………...…………………………………………………………………………..……..S14

**Fig. S13.** Baseline boxplot of K238G AeL at 10°C, pH 3, over a 100-minute detection……………………………………..…S15

**Fig. S14.** Glycopeptide-membrane interaction……………………………………………….….....................................................S16

**Fig. S15.** *I*_0_ histogram and selected range of *I*_0_…………………………………………………….……………......................................S17

**Fig. S16.** HDBSCAN cluster analysis of five types of truncated O-glycopeptides…………………………………………………....S18

**Fig. S17.** Sub-type classification of STF and STn peptides………………………….…………………………………………………….S19

**Fig. S18.** The performance of MLP model……………………………………………………..…….……………………….………………………S20

**Fig. S19.** The scatter plots of Tn peptide and the mixture of Tn and STn peptides in cell lysate. ……..…........................S21

**Fig. S20.** Typical current traces of K238G AeL detecting 1% BSA without Tn peptides and with Tn peptides………S22

**Fig. S21.** The scatter plots of K238G AeL detecting 1% BSA without Tn peptides and with Tn peptides……………S23

**Table S1.** The statistic of capture frequency of WT peptide detected by different nanopores………....... S24

**Table S2.** The statistic of capture frequency of WT and Tn peptides detected by K238G AeL in different voltages….S25

**Table S3.** The statistic of capture frequency of five types of truncated O-glycopeptides after HDBSCAN cluster analysis……………………………………………………………………………………………………..………………………………………………………….S26

**Table S4.** The statistical *I*/*I*_0_ of the O-glycopeptides in mixture……………………………………………………………………………….S27


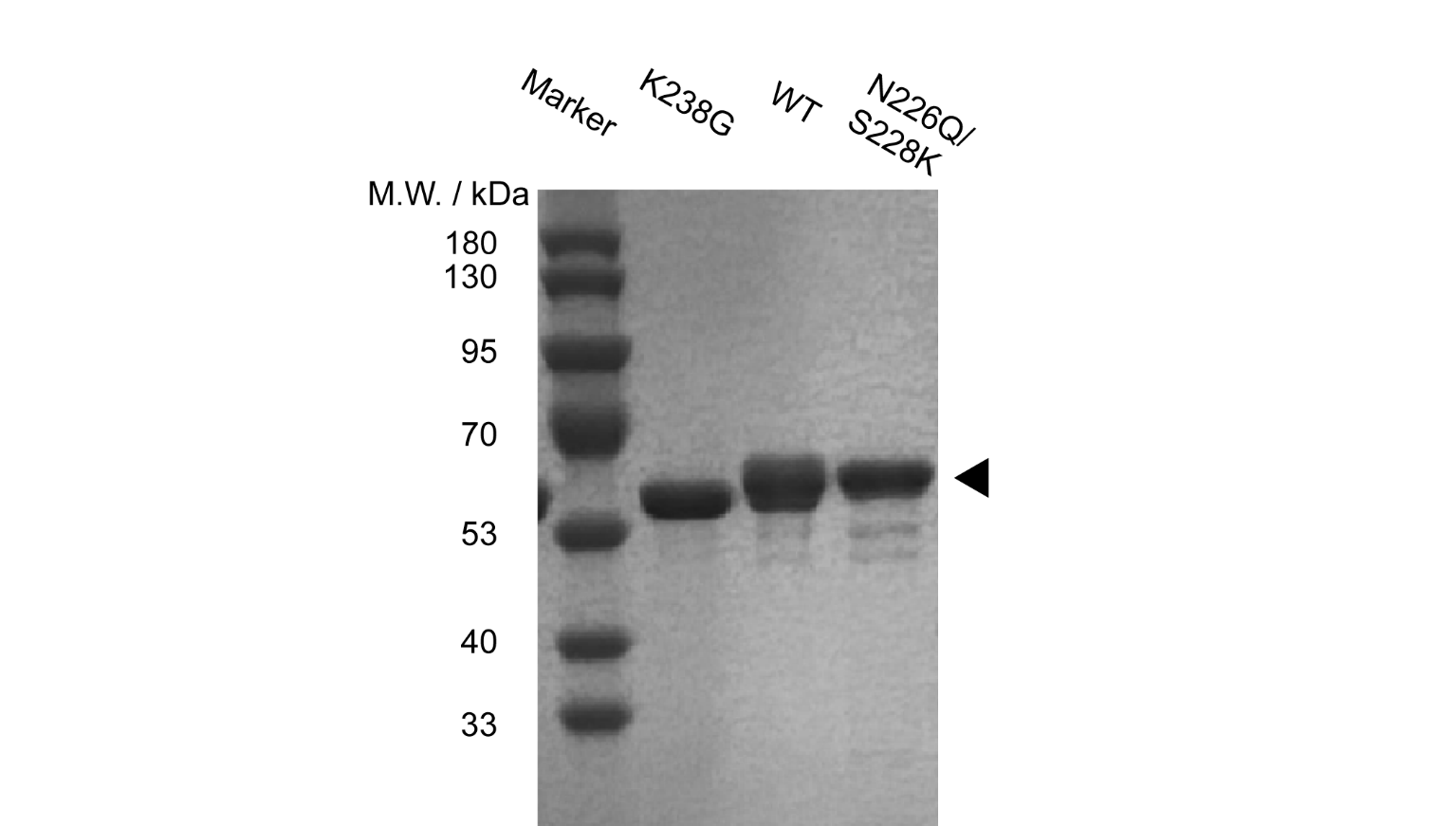


**Fig. S1.** SDS-PAGE analysis of the proaerolysin of K238G mutant, WT and the N226Q/S228K mutant (from left to right). The black triangle shows the target protein band.


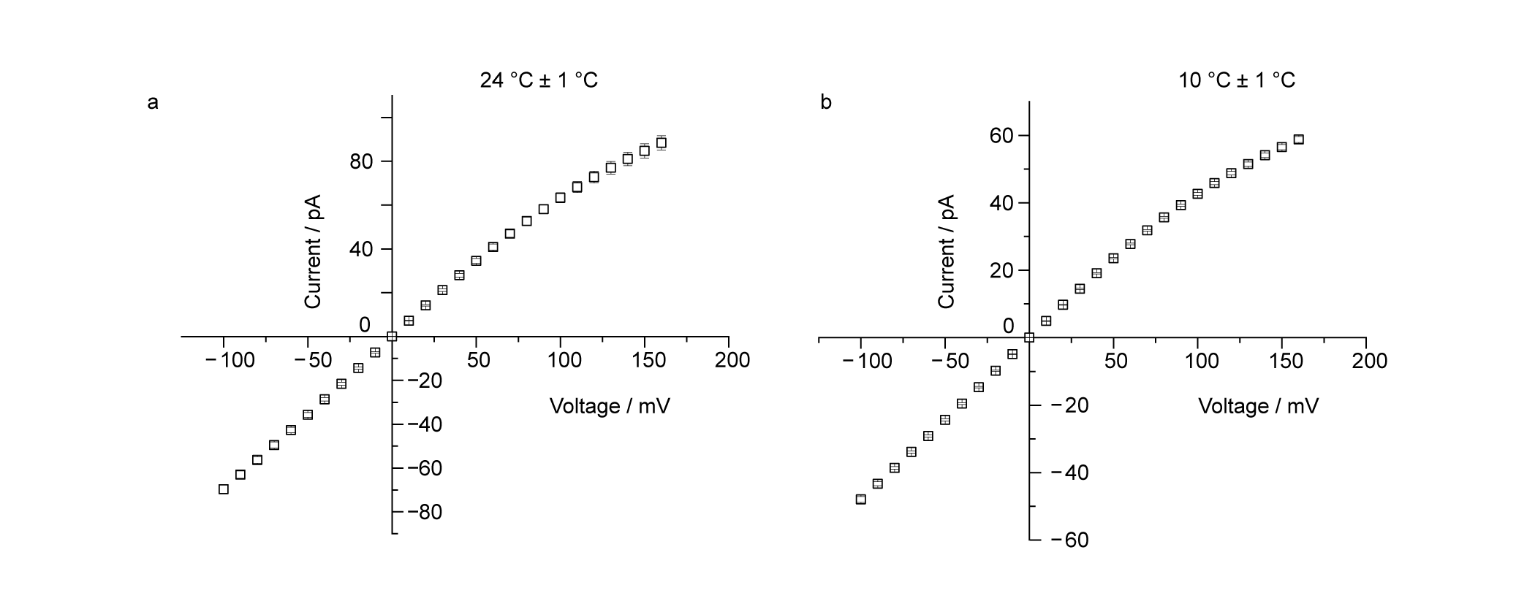


**Fig. S2.** *I*-*V* curves of the K238G AeL at 24 ± 1°C (a) and 10 ± 1°C (b). Data was obtained in the solution containing 1 M KCl at pH 3. The error bar comes from statistic of seven independent experiments in (a), and three independent experiments in (b).


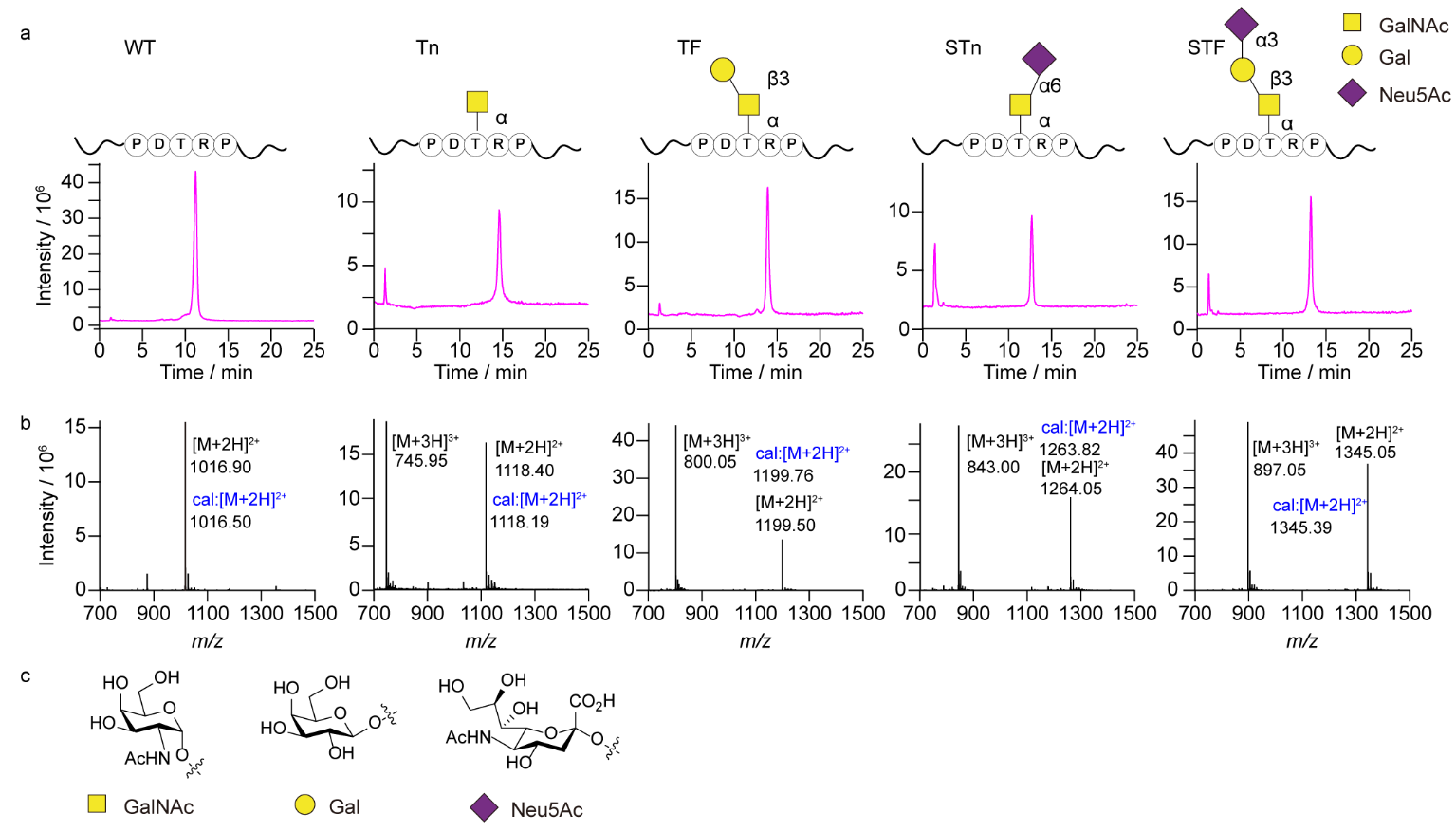


**Fig. S3.** The LC-MS characterization of O-glycopeptides. (a) Chromatogram and (b) ESI-MS spectra of O-glycopeptides for WT, Tn, TF, STn and STF (from left to right). Calculated [M+2H]^2+^ for WT, Tn, TF, STn, STF are 1016.50, 1118.19, 1199.76, 1263.82 ,1345.39, respectively.


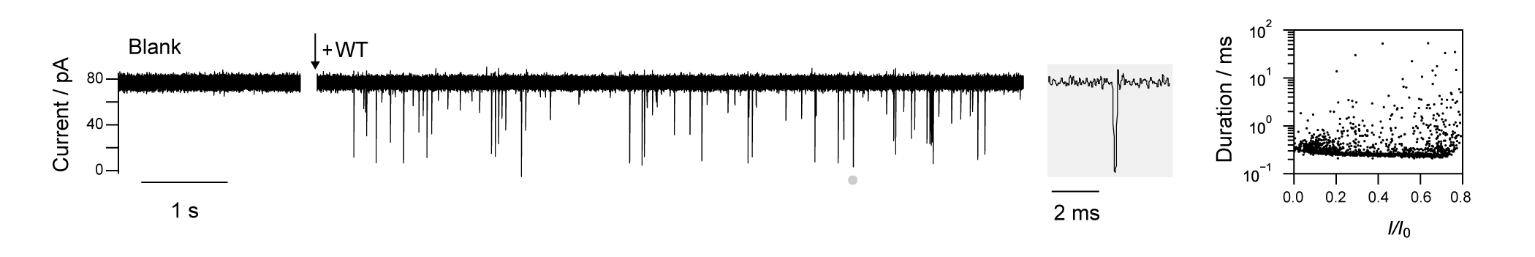


**Fig. S4.** Typical current trace (left) and the scatter plot of *I*/*I*_0_ vs duration of WT peptide (right) detected by N226Q/S228K AeL at 24 ± 1 °C and pH 7.4. The concentration of WT peptide is fixed at 10 μM. The experiments were performed in 1 M KCl, 10 mM Tris, under an applied voltage of +160 mV.


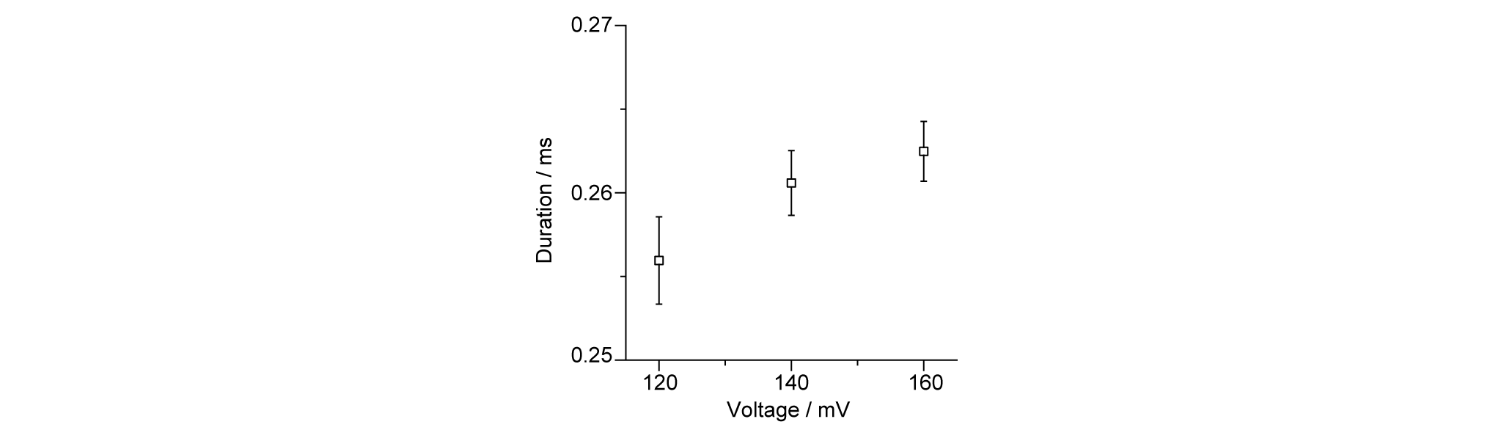


**Fig. S5.** The voltage dependency of duration for the WT peptide detected by N226Q/S228K AeL. The applied voltage is ranging from +120 mV to +160 mV, respectively. The concentration of WT peptide is fixed at 10 μM. Standard deviation(SD) from peak fit is used as the inferential error bar. The experiments were performed in 1 M KCl, 10 mM Tris, pH 7.4.


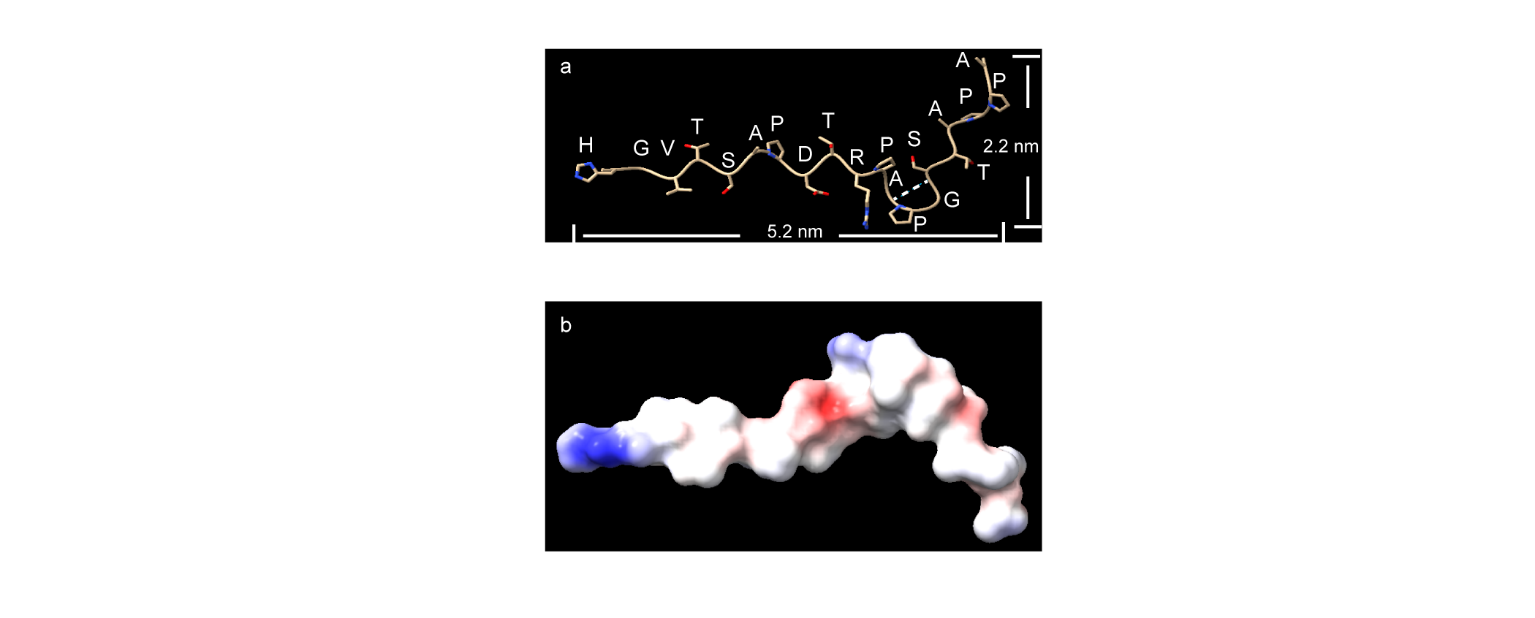


**Fig. S6.** The structure of WT peptide (sequence: N’-HGVTSAPDTRPAPGSTAPPA-C’). (a) The structure of WT peptide predicted by Alphafold2. The white dashed lines indicate potential hydrogen bonds. (b) Electrostatic potential map of the WT peptide. The potential values in the Fig. range from -0.477 to 0.466 eV at 25 °C.


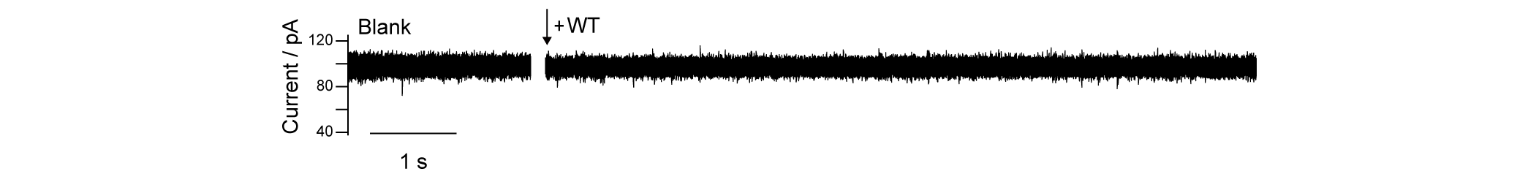


Fig. S7. Typical current trace of WT peptide detected by K238G AeL at 24 ± 1 °C and pH 7.4. The concentration of WT peptide is fixed at 10 μM. The experiment was performed in 1 M KCl, 10 mM Tris, under an applied voltage of +160 mV.

**
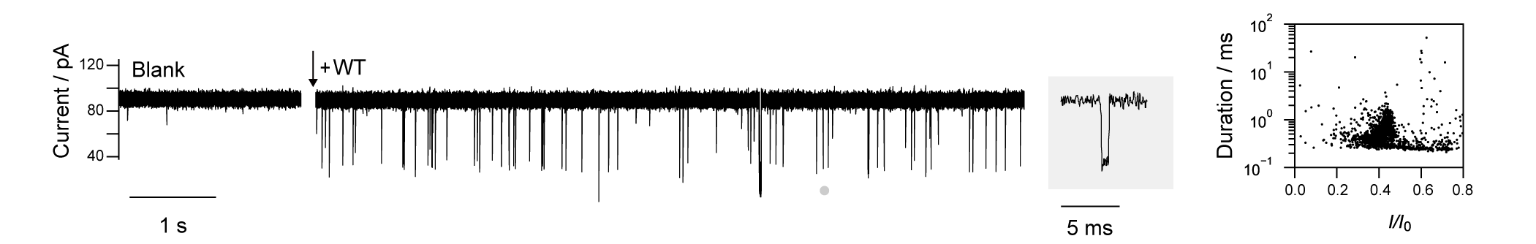
**

**Fig. S8.** Typical current trace (left) and the scatter plot of *I*/*I*_0_ vs duration of WT peptide (right) detected by K238G AeL at 24 ± 1 °C and pH 3. The concentration of WT peptide is fixed at 10 μM. The experiments were performed in 1 M KCl, 10 mM Tris, under an applied voltage of +160 mV.


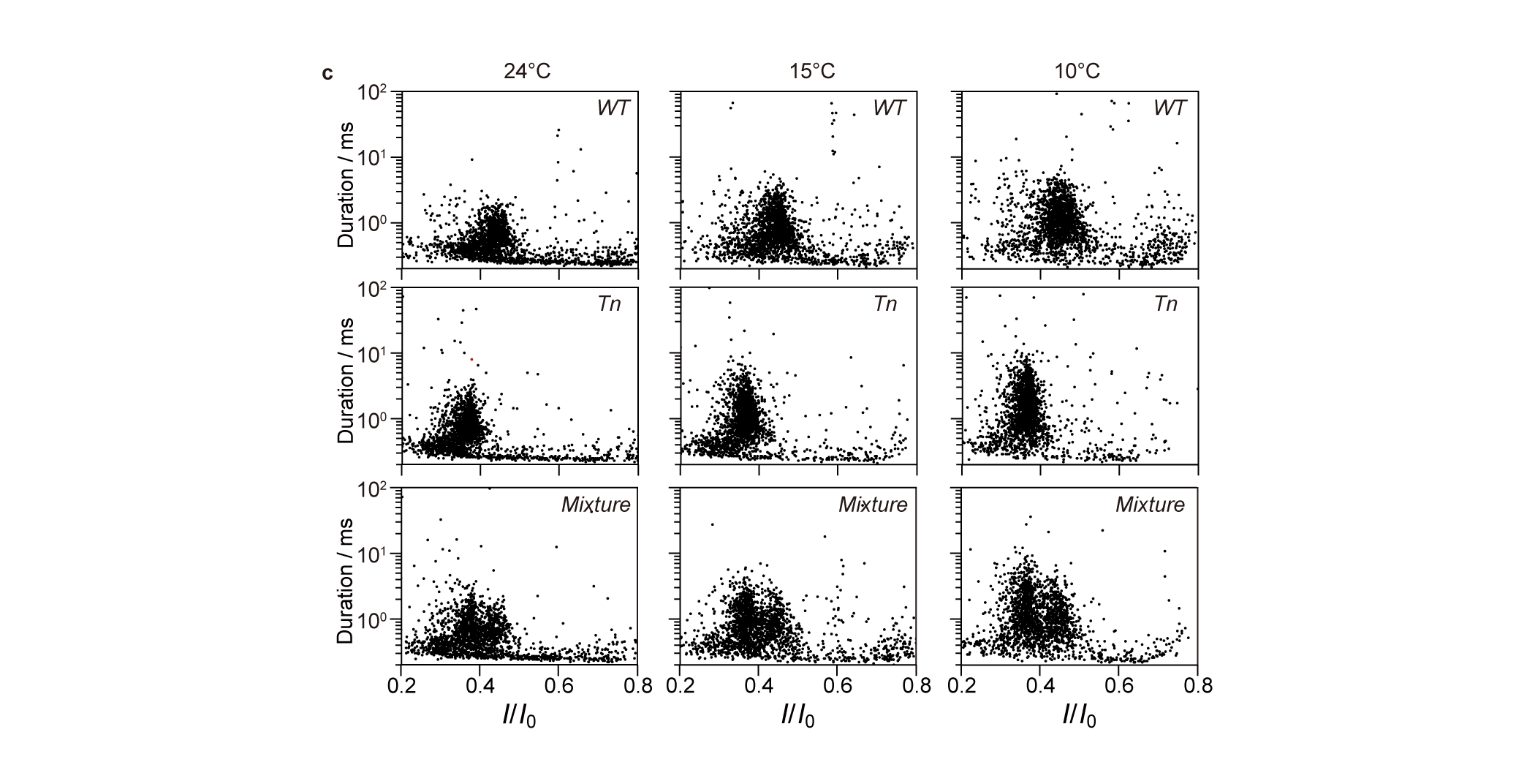


**Fig. S9.** *I/I_0_* vs duration scatter plot of WT, Tn peptides and their mixtures at different temperatures.


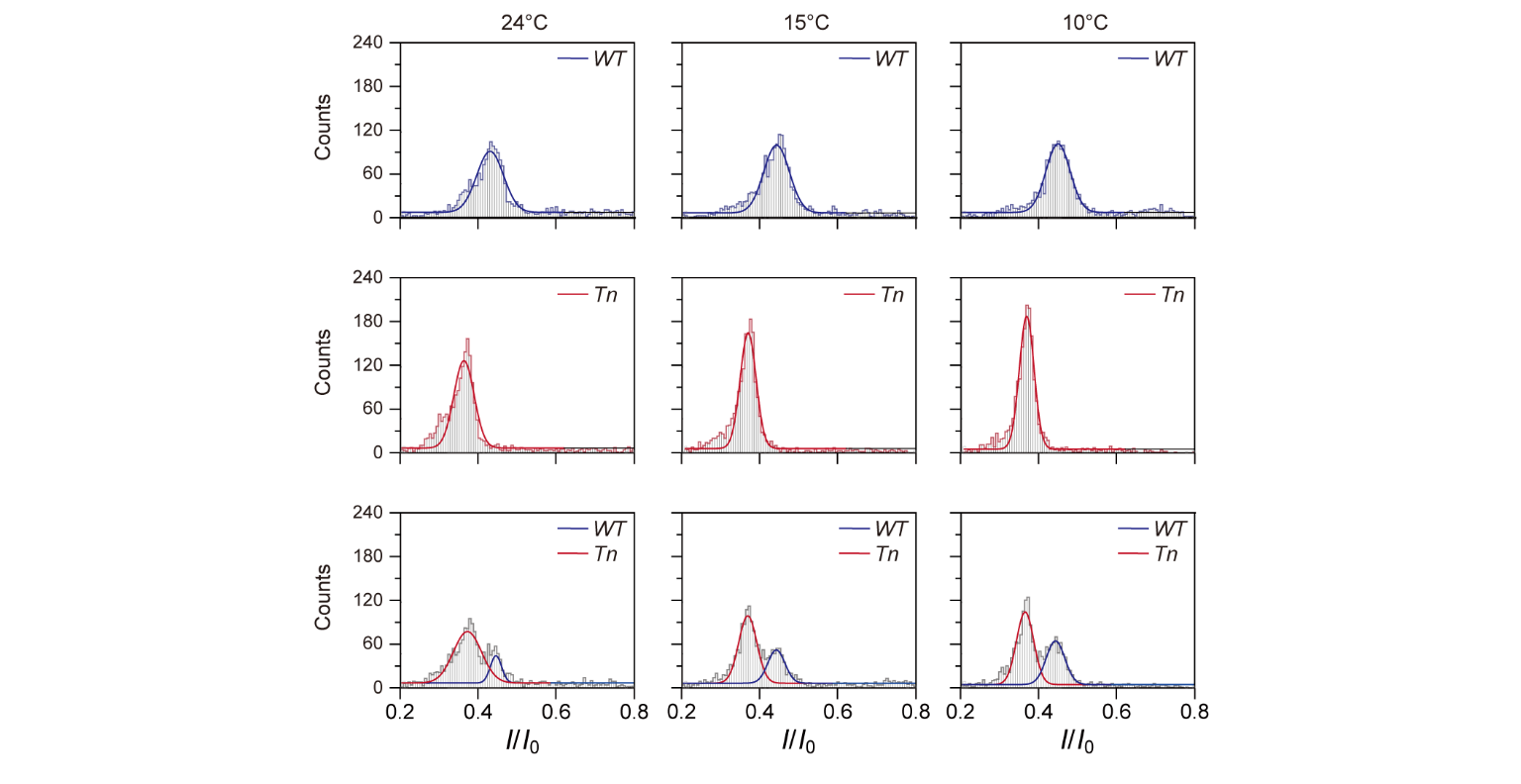


**Fig. S10.** *I/I_0_* histogram of WT, Tn peptides and their mixtures at different temperatures. The blue and red curve represents the Gaussian fitting to the *I*/*I*_0_ histograms for WT and Tn peptides, respectively.

**
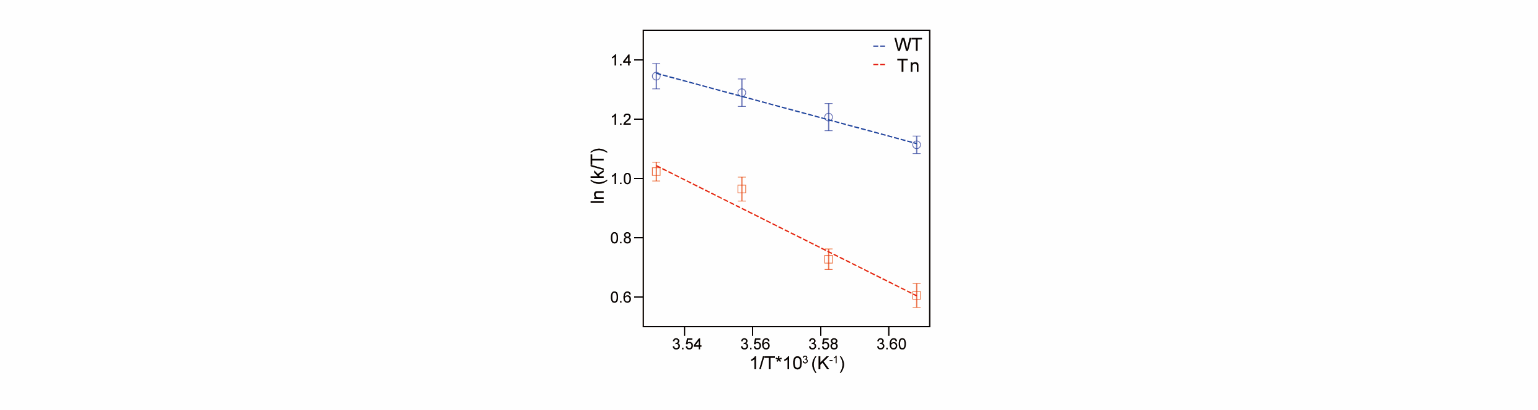
**

**Fig. S11.** The scatter plot of ln (k_off_/T) versus 1/T of the WT and Tn peptides, respectively. The dash lines represent the linear fitting curve.


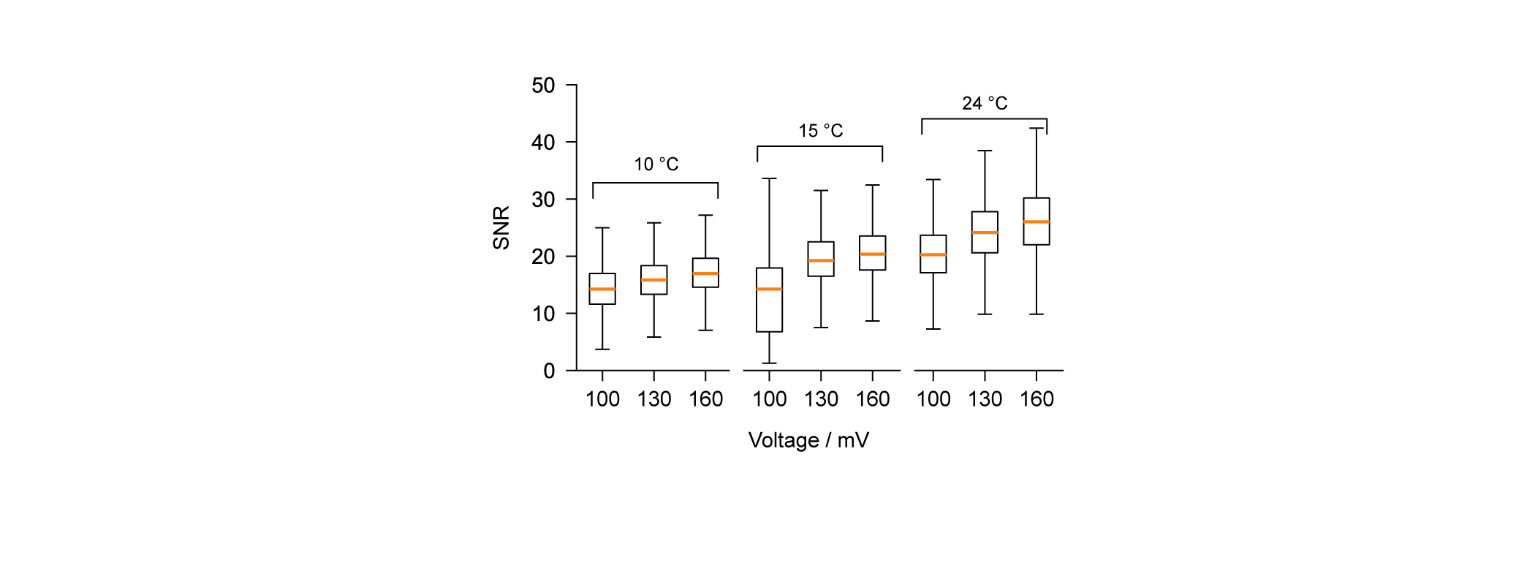


**Fig. S12.** Signal-noise ratio (SNR) of WT peptide detection using K238G AeL under different temperatures (10°C, 15°C, 24°C) and voltages (+100 mV, +130 mV, +160 mV). The SNR was calculated by dividing the Δ*I*/*I*_0_ by the SD of *I*_0_. The concentration of WT peptide is fixed at 10 μM. The experiments were performed in 1 M KCl, 10 mM Tris, pH 3, at 10 ± 1 °C. The upper and lower bounds of boxes show 25% and 75% percentiles of the data, respectively. The median of data is shown as the horizontal orange line in the box.


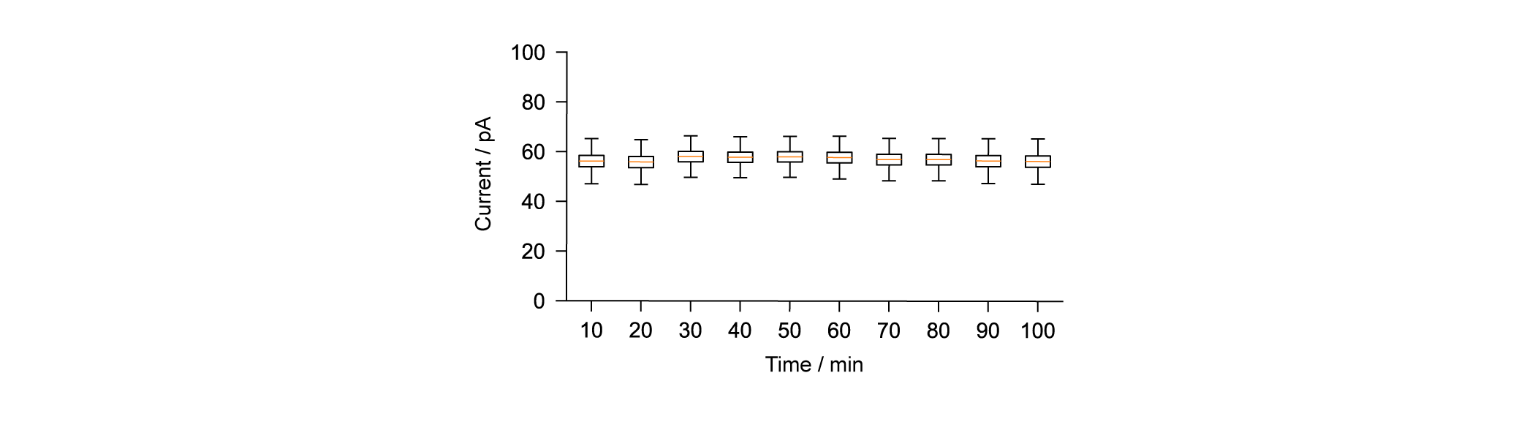


Fig. S13. Baseline boxplot of K238G AeL at 10°C and pH 3 over a period of 100 minutes. The data was sampled every 10 minutes. Each sample consists of a 10-second recording. The experiment was performed in 1 M KCl,10 mM Tris. The upper and lower bounds of boxes show 25% and 75% percentiles of the data, respectively. The median of data is shown as the horizontal orange line in the box. The error bar comes from the baseline noise. The baseline noise is defined as the peak-to-peak value of a number of consecutive point measurements of the background signal.


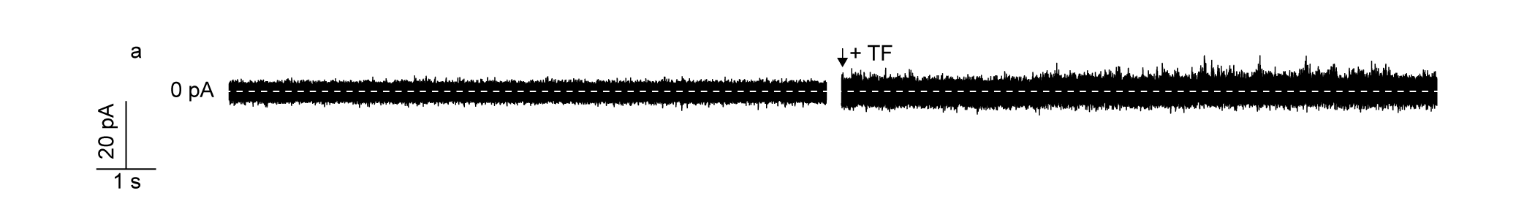


**Fig. S14.** Glycopeptide-membrane interaction of TF peptide. (a) the current trace of formed lipid bilayer before (left) and after addition (right) of TF peptide, in the absence of nanopore insertion. After addition of TF peptide, the SD of the current trace broadened and upward spikes appeared. This result suggested that TF could interact with the membrane. The experiments were performed in 1 M KCl, 10 mM Tris, pH 3, under an applied voltage of +160 mV at 10 ± 1 °C.


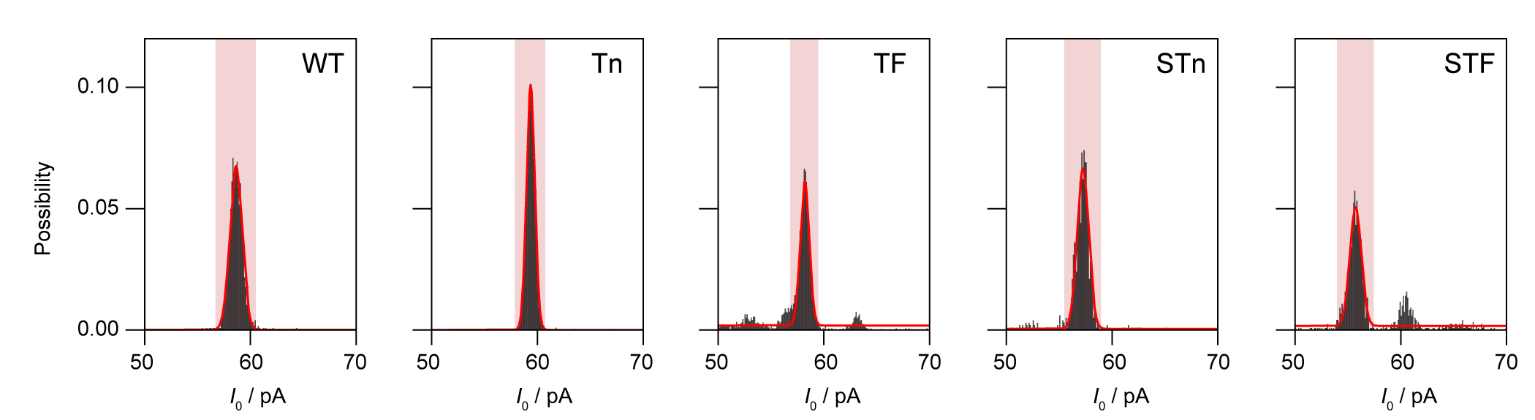


**Fig. S15.** The histogram of *I*_0_ values from each event. The red curve represents the Gaussian fitting to the *I*_0_ histogram. The red area represents the selected data which encompasses 99% of the fitted peak, ensuring that most of the relevant data points around the central distribution are included.


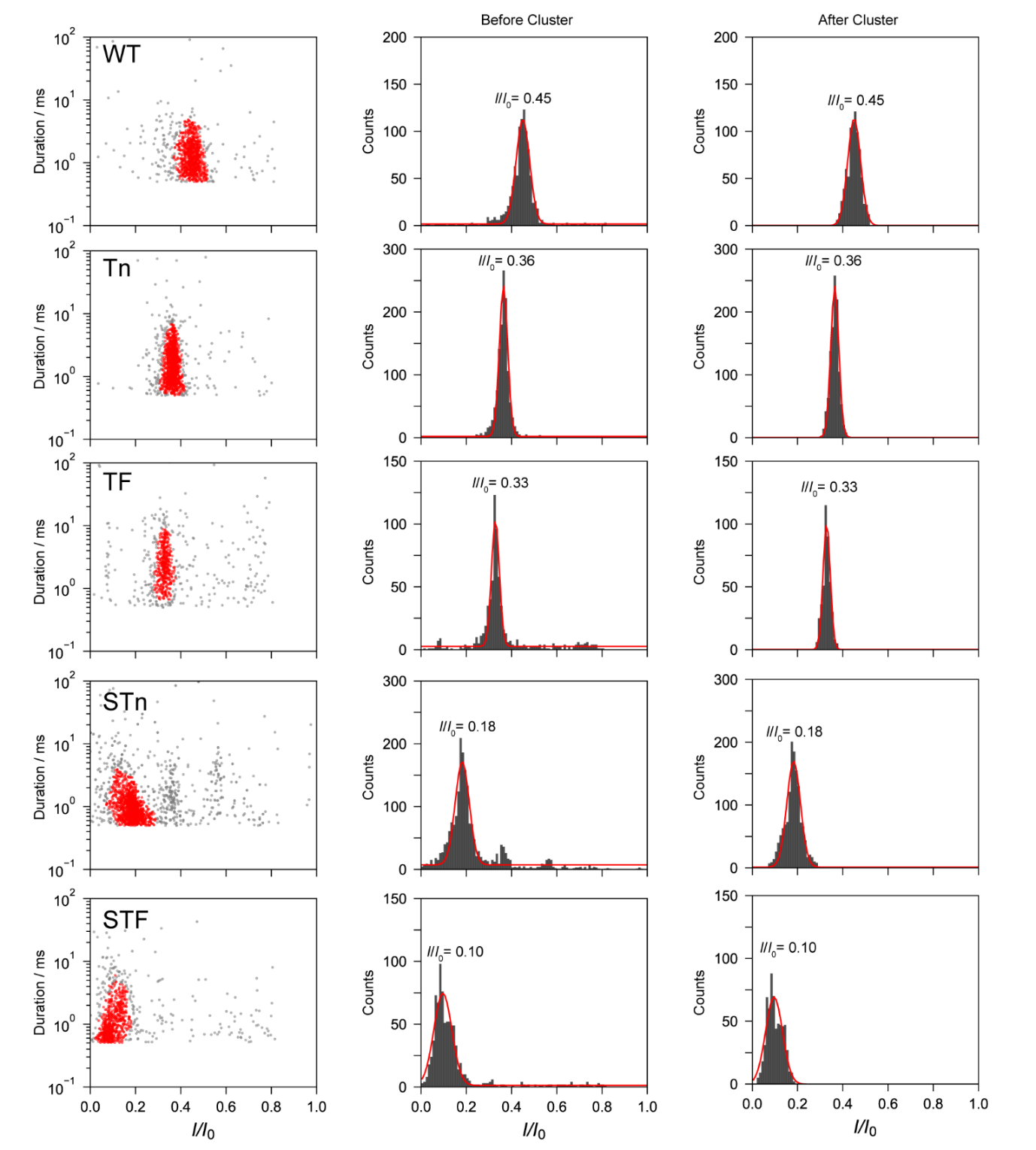


**Fig. S16.** HDBSCAN cluster analysis of five O-glycopeptides. From left to right: the scatter plots of *I*/*I*_0_ vs duration of the clustering results, the histograms of *I*/*I*_0_ before clustering, and the histograms of *I*/*I*_0_ after clustering. The red dots in the scatter plot refer to the major class that will be used for subsequent machine learning training, while the gray dots refer to the other class that will be removed. The data distribution remained nearly unchanged before and after clustering, ensuring the reliability of the analysis. The experiments were performed in 1 M KCl, 10 mM Tris, pH 3, under an applied voltage of +160 mV at 10 ± 1 °C.

**
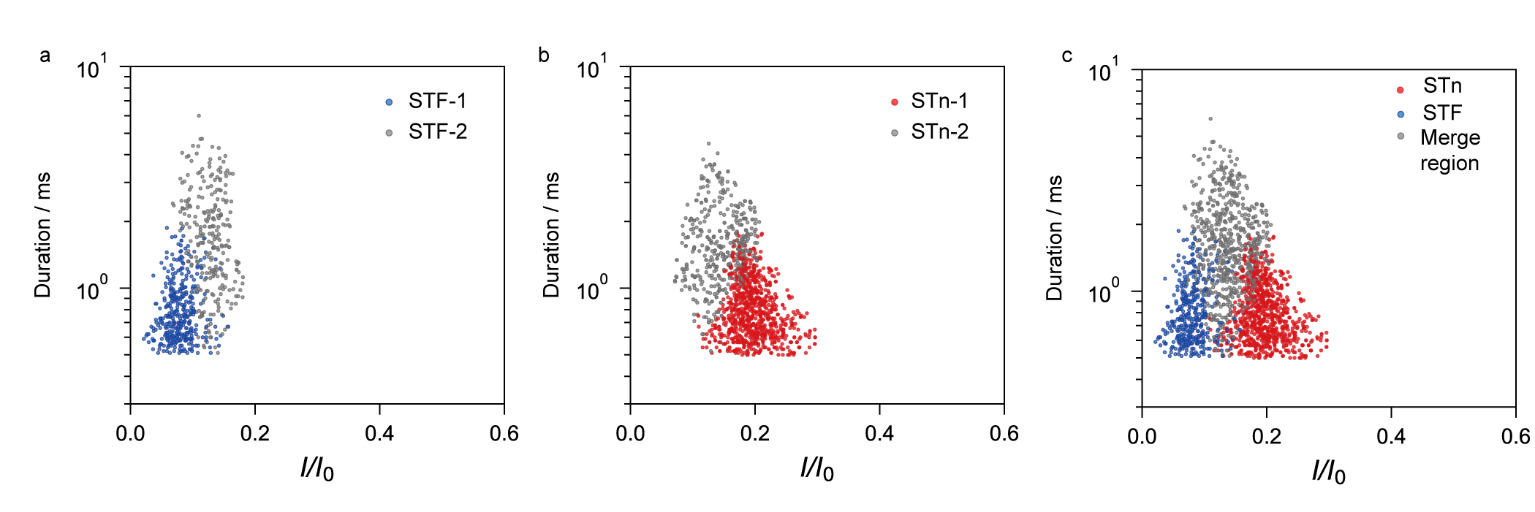
**

**Fig. S17.** Sub-type classification of (a) STF and (b) STn. Agglomerative Clustering method was used to cluster STF and STn events into two types based on the difference in duration time and *I*/*I*_0_. The major types of STF-1 and STn-1 events are distributed concentrically, whereas STF-2 and STn-2 events are more dispersed and overlapping with each other. (c) the merged scatter plot of scatter plots a and b.


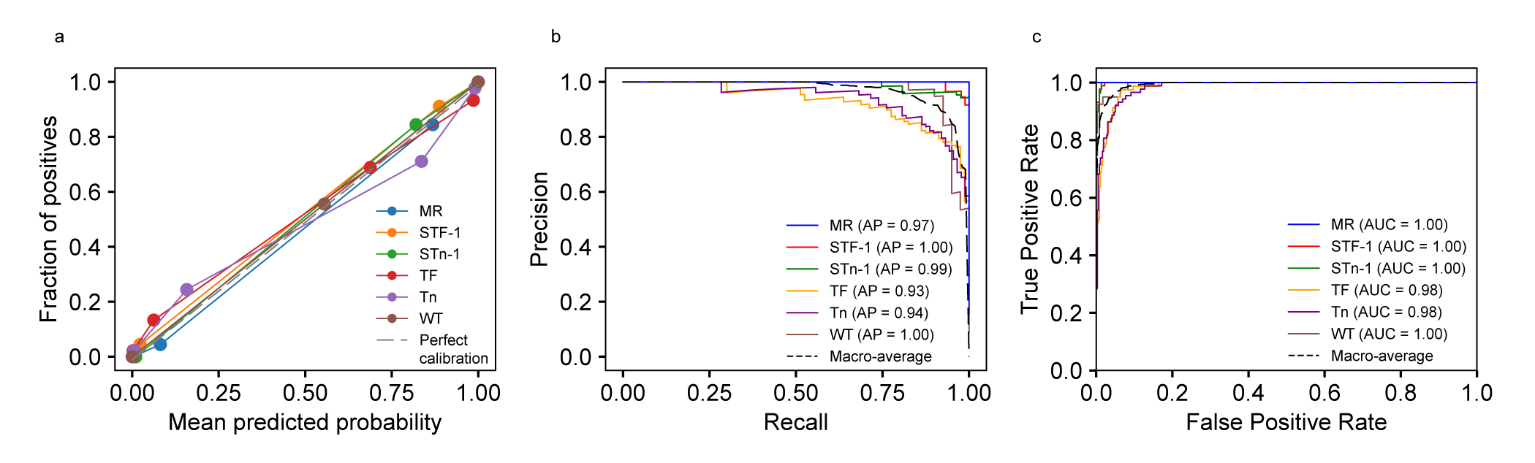


**Fig. S18.** (a) The calibration curve, (b) precision-recall curve and (c) ROC curve of MLP model.


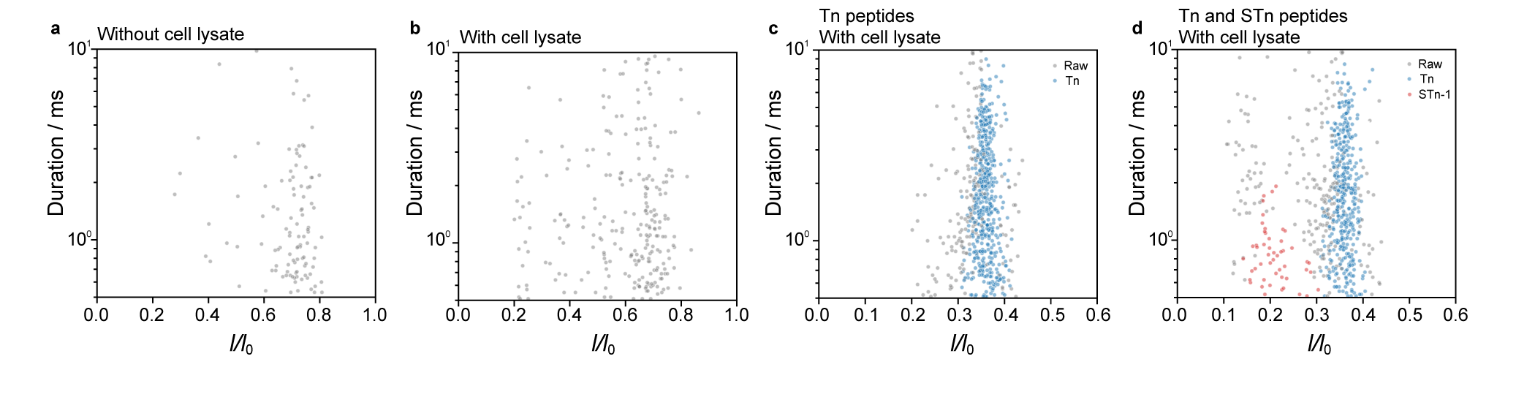
**Fig. S19.** The scatter plot of blank background (a), the cell lysate (b), STn peptides in cell lysate (c) and the mixture of STn and Tn peptides in cell lysate (d) detected by K238G aerolysin nanopore. The *I*/*I*_0_ value of most background events are higher than 0.6. The data of Tn peptide and the mixture (*I*/*I*_0_ < 0.5) were clustered to remove the outliers before the prediction of machine learning. The concentration of Tn and STn peptides are fixed at 5μM and 2μM respectively. The gray points refer to events before prediction. The blue points refer to Tn events and the red points refer to STn-1 events predicted by MLP model. The experiments were performed in 1 M KCl, 10 mM Tris, pH 3, containing 0.2% cell lysate, under an applied voltage of +160 mV at 10 ± 1 °C.

**
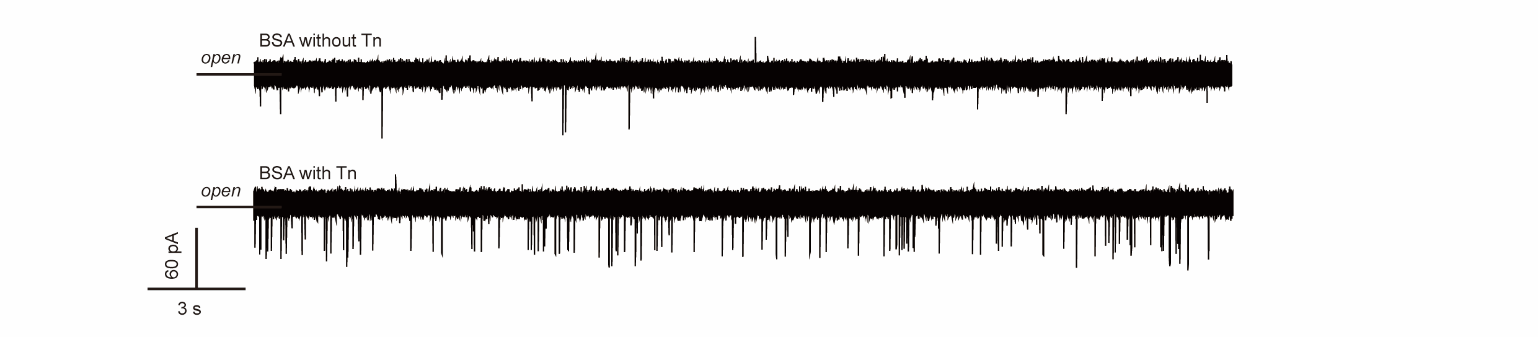
**

**Fig. S20.** Typical current traces of K238G AeL detecting 1% BSA without Tn peptides and with Tn peptides at 10 ± 1 °C and pH 3. The concentration of Tn peptide is fixed at 10 μM.

**
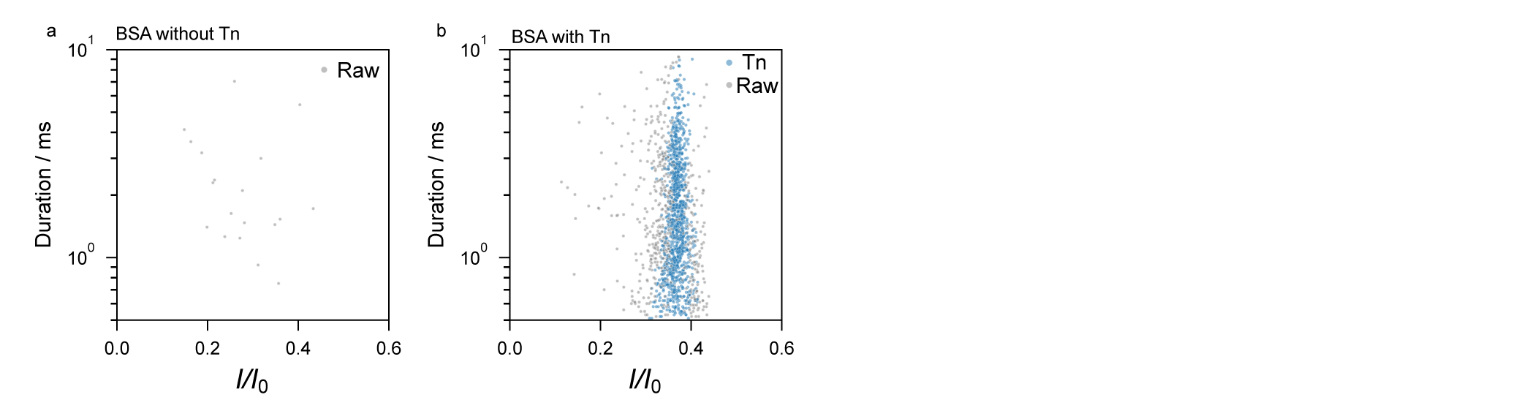
**

**Fig. S21.** The scatter plot of K238G AeL detecting (a) 1% BSA without Tn peptides and (b) with Tn peptides at 10 ± 1 °C and pH 3. The concentration of Tn peptide is fixed at 10 μM. The grey points represent the raw data (SD > 0), the blue points represent Tn peptides predicted by MLP model.

**Table S1.** The statistical capture frequency of WT peptide detected by AeL mutants.

| Nanopore^†^ | pH | Capture frequency  / μM^-1^min^-1 *^ |
| --- | --- | --- |
| N226Q/S228K | 7.4 | 11.6 ± 1.0 |
| K238G | 3.0 | 34.6 ± 2.1 |

^†^The measurement was in 1M KCl, 10 mM Tris, under an applied voltage of +160 mV, at 24 ± 1 °C.

^*^ (mean ± SD)

**Table S2.** The statistical capture frequency of the WT and Tn peptides detected by K238G AeL in different voltages.

| Peptides^†^ | Voltage / mV | Capture frequency^‖^  / μM^-1^min^-1 *^ |
| --- | --- | --- |
| WT | 100 | 3.9 ± 0.4 |
| WT | 130 | 9.5 ± 1.2 |
| WT | 160 | 14.7 ± 0.7 |
| Tn | 100 | 4.8 ± 0.7 |
| Tn | 130 | 11.2 ± 0.9 |
| Tn | 160 | 17.2 ± 1.1 |

^†^The measurement was in 1M KCl, 10 mM Tris, pH 3, at 10 ± 1 °C.

^‖^Capture frequency was calculated as the average number of events per minute from 300 randomly selected one-minute segments within the 10-minute recording.

^*^ (mean ± SD)

**Table S3.** The statistical I/I0 of the O-glycopeptides in mixture

| Peptides^†^ | Capture frequency  / μM^-1^min^-1 *^ |
| --- | --- |
| WT | 13.4 ± 0.6 |
| Tn | 15.1 ± 1.0 |
| TF | 25.0 ± 4.5 |
| STn | 5.8 ± 1.0 |
| STF | 4.2 ± 2.0 |

^†^The measurement was in 1M KCl, 10 mM Tris, pH 3, under an applied voltage of +160 mV, at 10 ± 1 °C.

^*^ (mean ± SD)

**Table S4.** The statistical *I*/*I*_0_ of the O-glycopeptides in mixture. ^ᵻ^

| Peptides | *I*/*I*_0_ |
| --- | --- |
| WT | 0.44 ± 0.03 |
| Tn | 0.37 ± 0.02 |
| TF | 0.32 ± 0.02 |
| STn | 0.20 ± 0.03 |
| STF | 0.11 ± 0.02 |

^ᵻ^ The MLP model was used to predict the blockade ratio (*I*/*I*₀) for each peptide in the mixture. Error bars represent errors obtained from Gauss fitting.
